# An autophagy-related gene expression signature for survival prediction in multiple cohorts of hepatocellular carcinoma patients

**DOI:** 10.18632/oncotarget.24089

**Published:** 2018-01-09

**Authors:** Peng Lin, Rong-Quan He, Yi-Wu Dang, Dong-Yue Wen, Jie Ma, Yun He, Gang Chen, Hong Yang

**Affiliations:** ^1^ Department of Medical Ultrasonics, First Affiliated Hospital of Guangxi Medical University, Nanning, Guangxi Zhuang Autonomous Region 530021, P. R. China; ^2^ Department of Medical Oncology, First Affiliated Hospital of Guangxi Medical University, Nanning, Guangxi Zhuang Autonomous Region 530021, P. R. China; ^3^ Department of Pathology, First Affiliated Hospital of Guangxi Medical University, Nanning, Guangxi Zhuang Autonomous Region 530021, P. R. China

**Keywords:** prognosis index, HCC, survival, autophagy

## Abstract

Prognostic signatures have been proposed as clinical tools to estimate prognosis in hepatocellular carcinoma (HCC), which is the second most common contributor to cancer-related death at present globally. Autophagy-related genes play a dynamic and fundamental role in HCC, but knowledge of their utility as prognostic markers is limited. Here, we facilitated univariate and multivariate Cox proportional hazards regression analyses to reveal that 3 autophagy-related genes (BIRC5, FOXO1 and SQSTM1) were closely related to the survival of HCC. Then, we generated a prognosis index (PI) for predicting overall survival (OS) based on the three genes, which was an independent prognostic indicator for the OS of HCC (HR = 1.930, 95% CI: 1.200–3.104, *P* = 0.007). The PI showed moderate performance for predicting the survival of HCC patients and its efficacy was validated by data from three microarrays (GSE10143, GSE10186 and GSE17856). Furthermore, we deeply mined the integrated large-scale datasets from public microarrays and immunohistochemistry to validate the overexpression of BIRC5 and SQSTM1 while down-regulated FOXO1 expression in HCC. Bioinformatic analysis offered the hypothesis that proliferative signals in high-risk HCC patients were disturbing and thereby facilitated inferior clinical outcomes. Collectively, the prognostic signature we proposed is a promising biomarker for monitoring outcome of HCC. Nevertheless, prospective experimental studies are needed to validate the clinical utility.

## INTRODUCTION

Hepatocellular carcinoma (HCC), the predominant primary tumor of the liver, is the second most common contributor to cancer-related death at present globally [1–3]. The considerably high mortality rate of liver tumor seriously threatens humanity, where 40,710 of the estimated new liver cancer cases in the United States in 2017 [4]. Although surgical resection technology or transplantation rapidly improved, the 5-year survival rate of HCC patients remains relatively low [5, 6]. The dismal clinical outcome of HCC is in part driven by the delay in diagnosis and the still-rudimentary prognosis monitoring [7]. Hepatocarcinogenesis is heterogeneous and contains a multi-step process, including genetic and epigenetic factors that forms its unique molecular fingerprint. Therefore, extensive analyses for identifying reliable biomarkers with prognostic significance that target major oncogenes in HCC is imperative.

Autophagy is a fundamental cell-physiologic regulator to ensure intracellular quality control, similar to apoptosis, energy production and waste removal [8, 9]. Given the prominent functions of autophagy, it is closely associated with diverse human pathologies, such as immune disorders [10, 11], neurodegenerative diseases [12, 13] and cancers [14, 15]. Oftentimes, compared with their normal counterparts, tumor cells are metabolically reprogrammed when several growth-promoting pathways are disturbed to obtain sufficient energy or additional stimuli to master of their own destinies. Interestingly, autophagy probably is present in both cancer and in cancer prevention, as well as potentially contributing to the growth of cancer. Additionally, its roles during the course of various tumors progression, including HCC [16], are variable. However, subtle mechanisms of autophagy in HCC are perplexing and have not been fully understood until now [17]. For the dynamic and fundamental role of autophagy in HCC, the prognostic signatures and therapeutic targets of autophagy-related genes could show promise for understanding the genetic control mechanisms of HCC and offering promising targets. Recently, several studies have been proposed to identify autophagy-related prognostic signature in pancreatic ductal adenocarcinoma [18] and gliomas [19]. However, potential autophagy-related prognostic biomarkers for HCC are still urgently needed.

Due to the recent advances in RNA sequencing (RNA-Seq) technology and several public databases, such as The Cancer Genome Atlas (TCGA, https://cancergenome.nih.gov/) and Gene Expression Omnibus (GEO, http://www.ncbi.nlm.nih.gov/geo/), it is feasible to identify several genes that can predict the clinical outcomes of HCC patients based on the expression profiles. In the present study, we first extracted autophagy-related genes from The Human Autophagy Database (HADb, http://www.autophagy.lu/index.html), which provides an informative and up-to-date list of human genes involved in autophagy directly or indirectly. [20] Additionally, we calculated and obtained a series of differentially expressed autophagy-related genes in HCC and elucidated the cellular and molecular characteristics, dynamic role and pathways of these genes in HCC. The highlight was that we placed special emphasis on the prognostic value of autophagy-related genes and constructed a specific prognosis index (PI) that accurately predicted the clinical outcomes of HCC patients. These results were evidently confirmed by comprehensive data sources. Through these means, we proposed the rationale for clinically available prognosis monitoring and new insights into the molecular mechanisms for HCC patients, with particular emphasis on autophagy.

## RESULTS

### Differentially expressed autophagy-related genes in HCC

**Figure 1:**
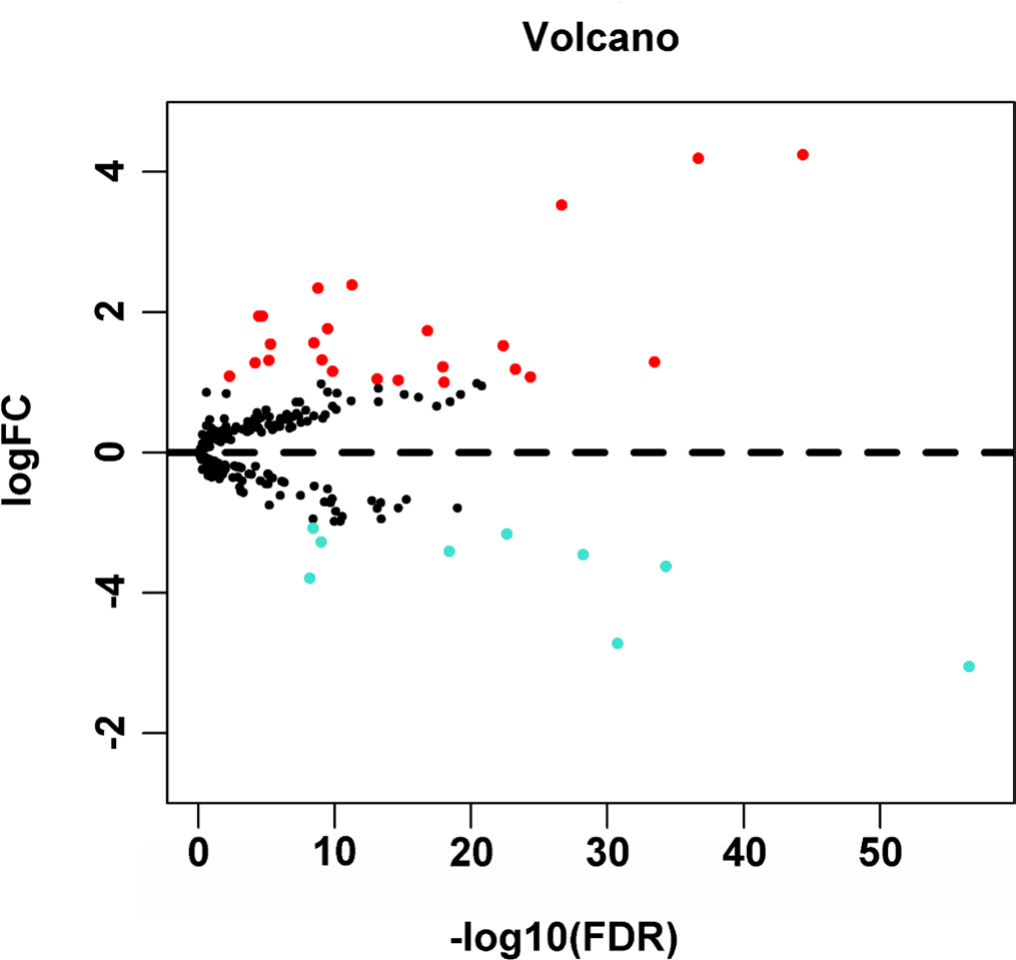
Volcano plot of the differentially expressed autophagy-related genes between HCC and non-tumor tissues Red dots indicated autophagy-related genes which were high expression in HCC and blue for low expression. This volcano plot was drawn by the ggplot2 package of R language.

**Figure 2:**
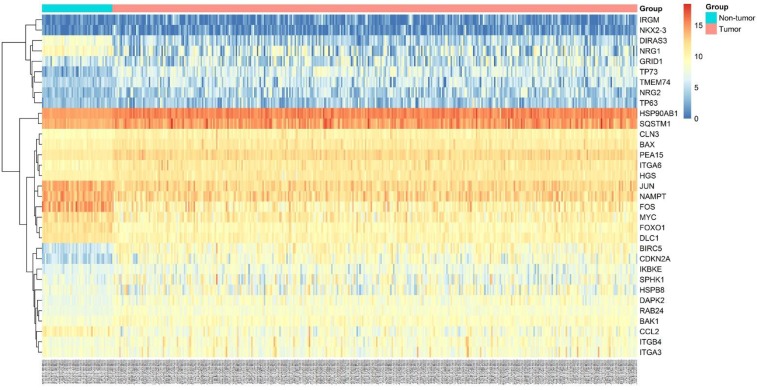
Heatmaps of 33 differently expressed autophagy-related genes Heatmap displayed the expression level of 33 differently expressed autophagy-related genes between HCC and non-tumor tissues. The brighter nodes indicated higher gene expression value while the darker indicated the lower gene expression value.

**Figure 3:**
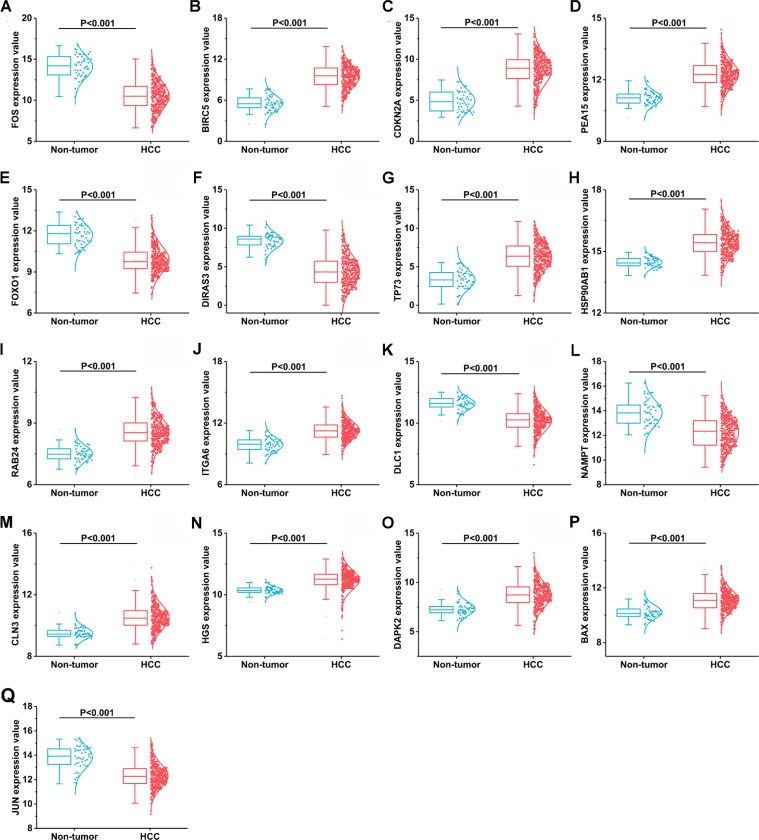
17 differentially expressed autophagy-related genes based on TCGA databases (**A**) FOS, (**B**) BIRC5, (**C**) CDKN2A, (**D**) PEA15, (**E**) FOXO1, (**F**) DIRAS3, (**G**)TP73, (**H**)HSP90AB1, (**I**) RAB24, (**J**) ITGA6, (**K**) DLC1, (**L**) NAMPT, (M) CLN3, (**N**) HGS, (**O**) DAPK2, (**P**) BAX, (**Q**) JUN.

**Figure 4:**
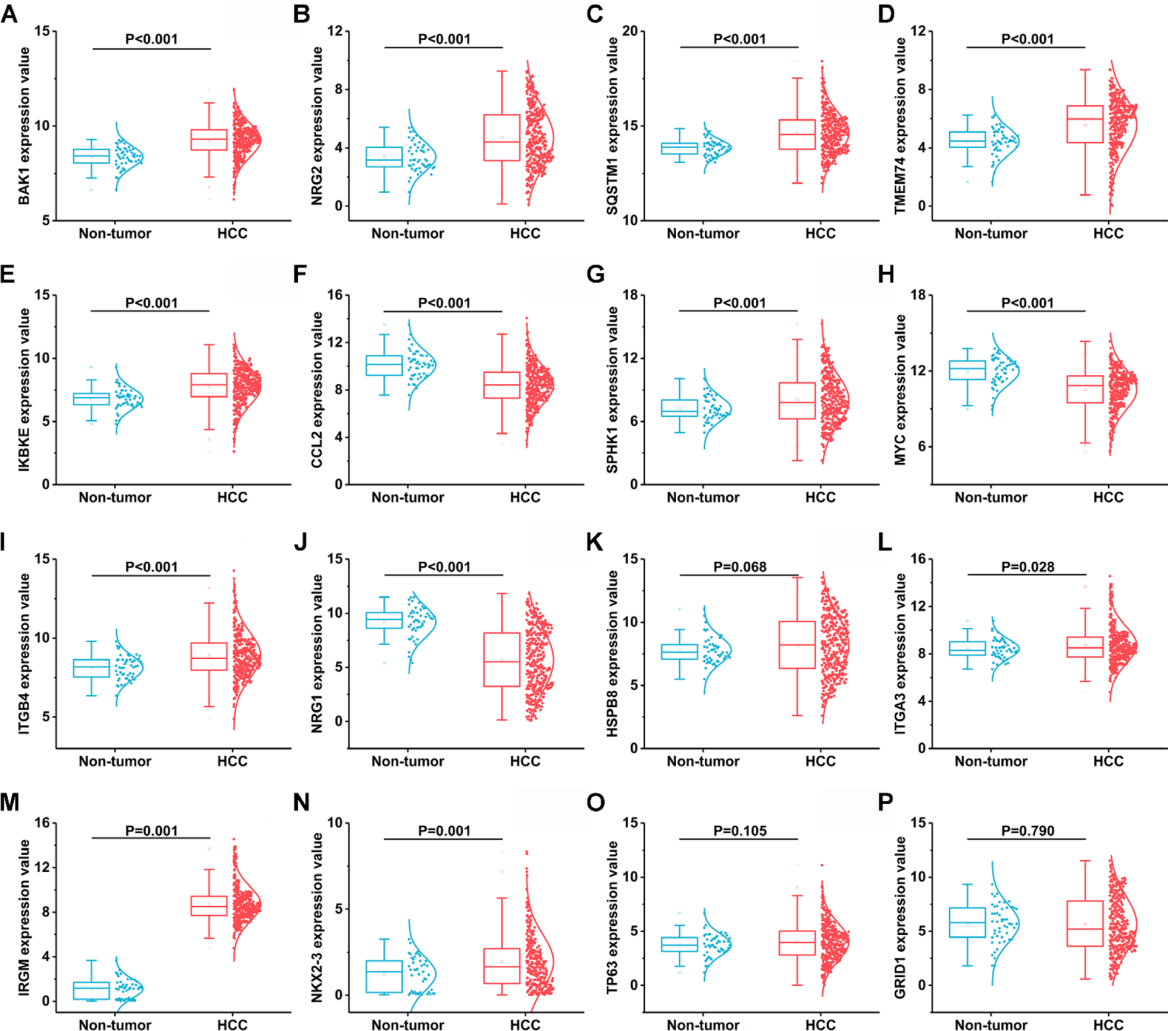
Another 16 differentially expressed autophagy-related genes based on TCGA databases (**A**) BAK1, (**B**) NRG2, (C) SQSTM1, (**D**) TMEM74, (**E**) IKBKE, (**F**) CCL2, (**G**) SPHK1, (**H**) MYC, (**I**) ITGB4, (**J**) NRG1, (**K**) HSPB8, (**L**) ITGA3, (**M**) IRGM, (**N**) NIKX2-3, (**O**) TP63, (**P**) GRID1.

**Figure 5:**
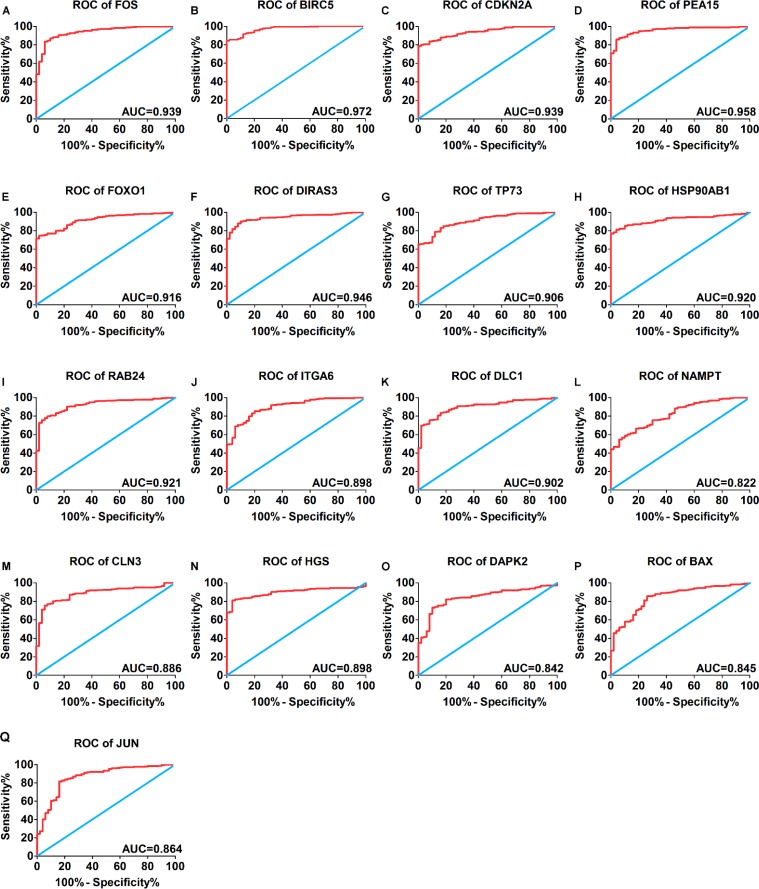
Receiver operating characteristic curves of 17 differentially expressed autophagy-related genes expression for the differentiation of HCC from non-tumor tissues based on TCGA (**A**) FOS, (B) BIRC5, (**C**) CDKN2A, (**D**) PEA15, (**E**) FOXO1, (**F**) DIRAS3, (**G**)TP73, (**H**)HSP90AB1, (**I**) RAB24, (**J**) ITGA6, (**K**) DLC1, (**L**) NAMPT, (**M**) CLN3, (N) HGS, (**O**) DAPK2, (**P**) BAX, (**Q**) JUN.

**Figure 6:**
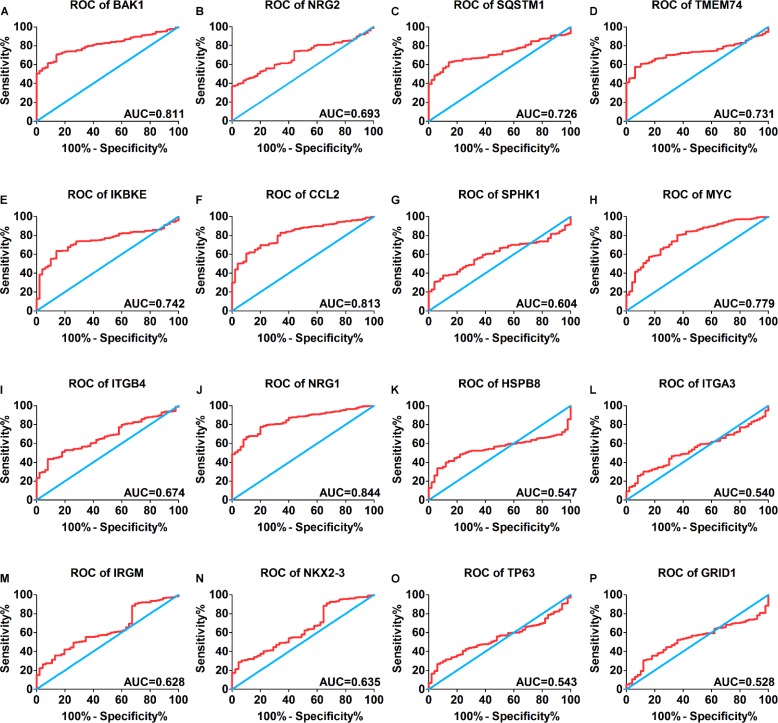
Receiver operating characteristic curves of another 16 differentially expressed autophagy-related genes expression for the differentiation of HCC from non-tumor tissues based on TCGA (**A**) BAK1, (**B**) NRG2, (**C**) SQSTM1, (**D**) TMEM74, (**E**) IKBKE, (**F**) CCL2, (**G**) SPHK1, (**H**) MYC, (**I**) ITGB4, (**J**) NRG1, (**K**) HSPB8, (**L**) ITGA3, (**M**) IRGM, (**N**) NIKX2-3, (**O**) TP63, (**P**) GRID1.

A total of 234 genes involved directly or indirectly in autophagy were downloaded via the online database HADb. After extracting expression data of these 234 autophagy-related genes from TCGA, 33 differentially expressed genes in HCC were identified (Figures 1 and 2), which were used for further investigation on their prognostic value. The independent sample *t*-test demonstrated that 21 genes showed remarkably higher expressions than non-cancerous tissues, including BIRC5, CDKN2A, PEA15, TP73, HSP90AB1, RAB24, ITGA6, CLN3, HGS, DAPK2, BAX, BAK1, NRG2, SQSTM1, TMEM74, IKBKE, SPHK1, ITGB4, ITGA3, IRGM and NKX2-3. Conversely, 9 genes (FOS, FOXO1, DIRAS3, DLC1, NAMPT, JUN, CCL2, MYC and NRG1) had notably lower expressions than the adjacent tissues. (Figures 3 and 4). Due to the discrepancy in calculation, no statistical significance was observed in HSPB8, TP63 and GRID1. Subsequently, ROC analysis was conducted to further investigate the ability of these genes to distinguish cancerous tissues from the non-cancerous ones. The results showed that the AUC value of 24 genes was above 0.7 (Figures 5–6), which confirmed that these genes excelled at differentiating between cancerous and non-cancerous tissues.

### Gene-enrichment and functional annotation for differentially expressed autophagy genes in HCC

**Figure 7:**
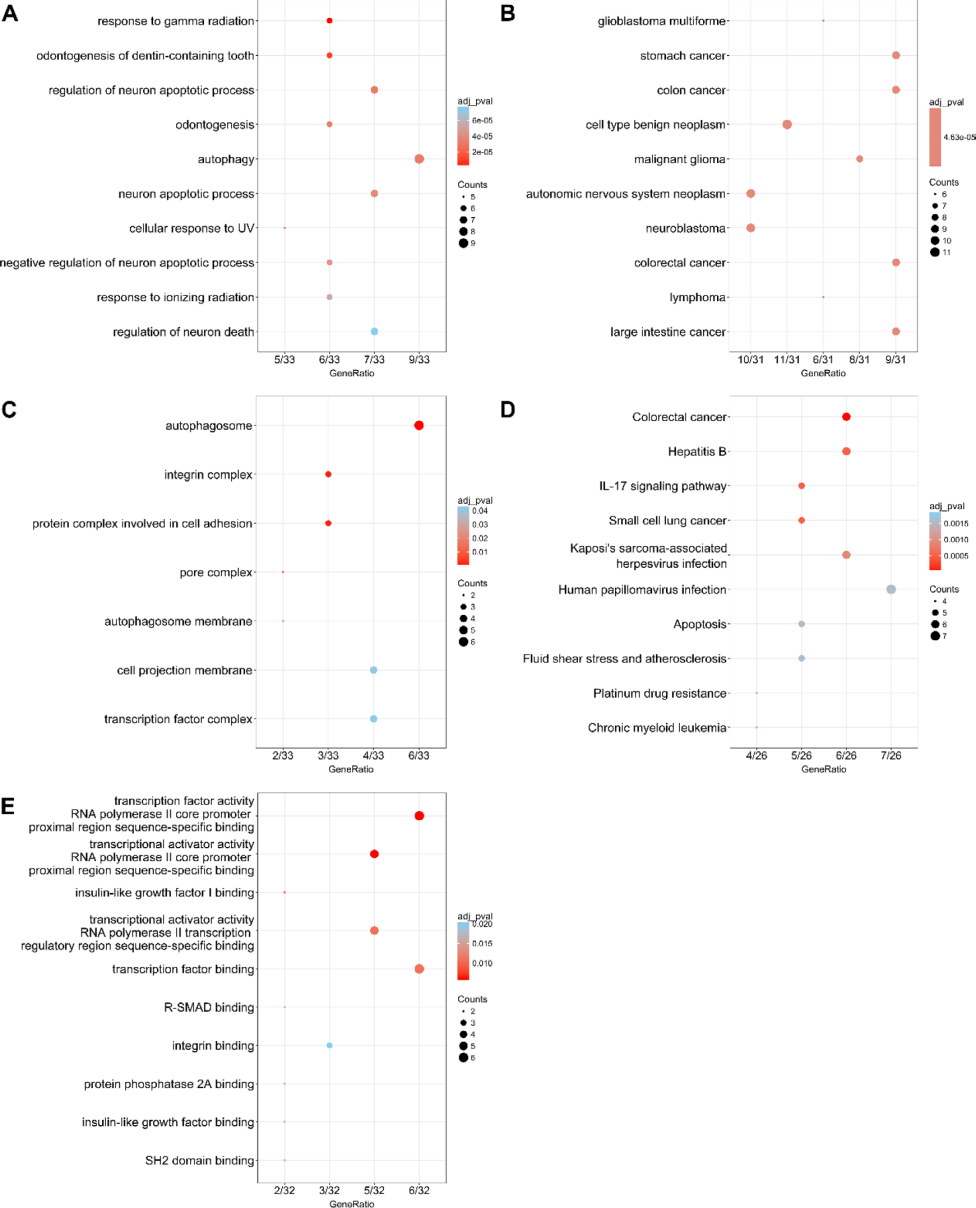
Dot plot of functional enrichment analyses (**A**) Biological process; (**B**) Disease ontology; (**C**) Cellular component; (**D**) Kyoto Encyclopedia of Genes and Genomes; (**E**) Molecular function.

**Figure 8:**
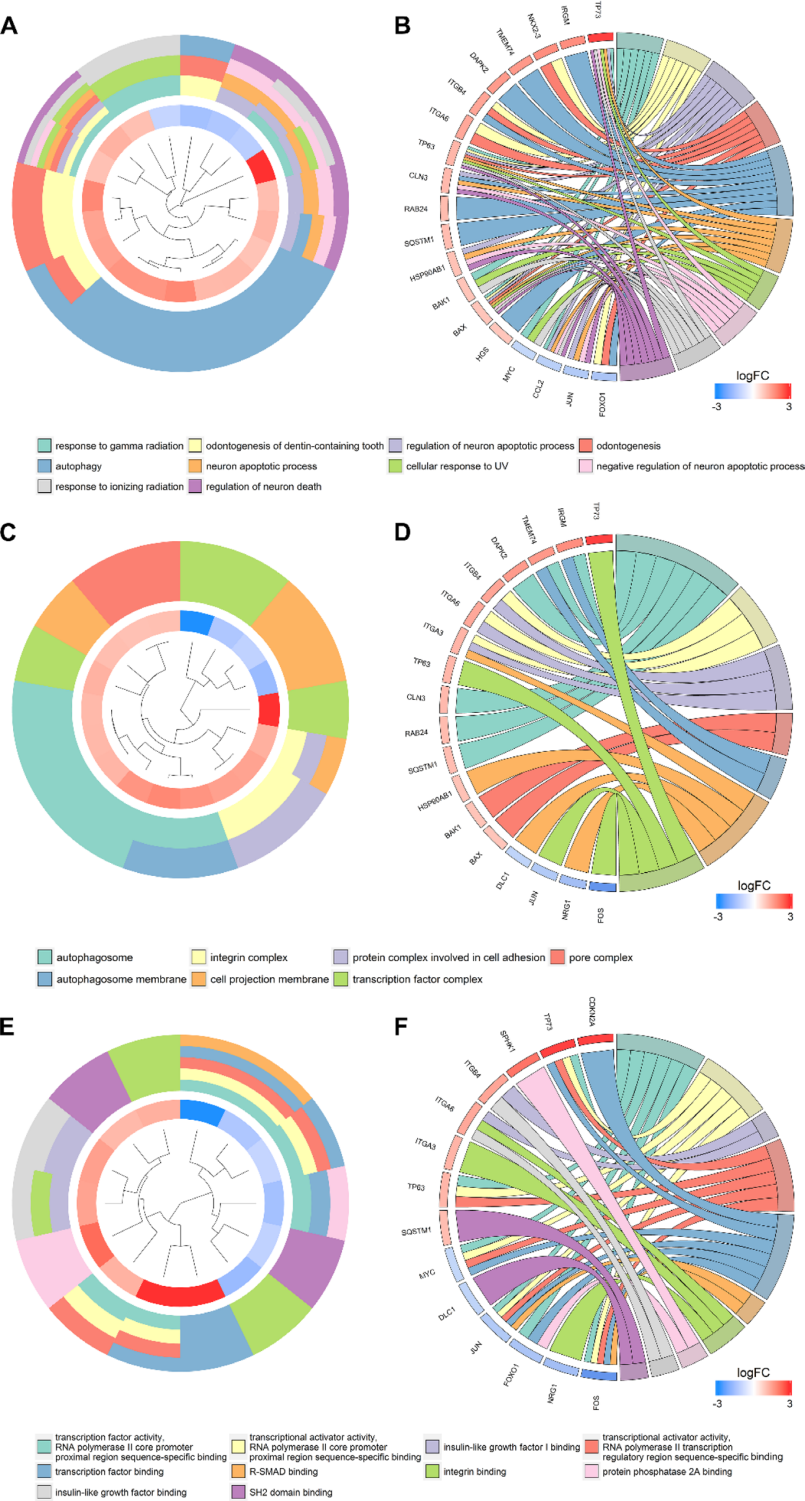
Gene ontology analysis of the differentially expressed genes Circle plot displaying a circular dendrogram of the clustering of the expression profiles. The inner ring shows the color-coded logFC, the outer ring the assigned biological process (**A**), cellular component (**C**) and molecular function (**E**) terms. Cluster displaying these genes are linked via ribbons to their assigned biological process (**B**), cellular component (**D**) and molecular function (**F**) terms. Blue-to-red coding next to the selected genes indicates logFC.

**Figure 9:**
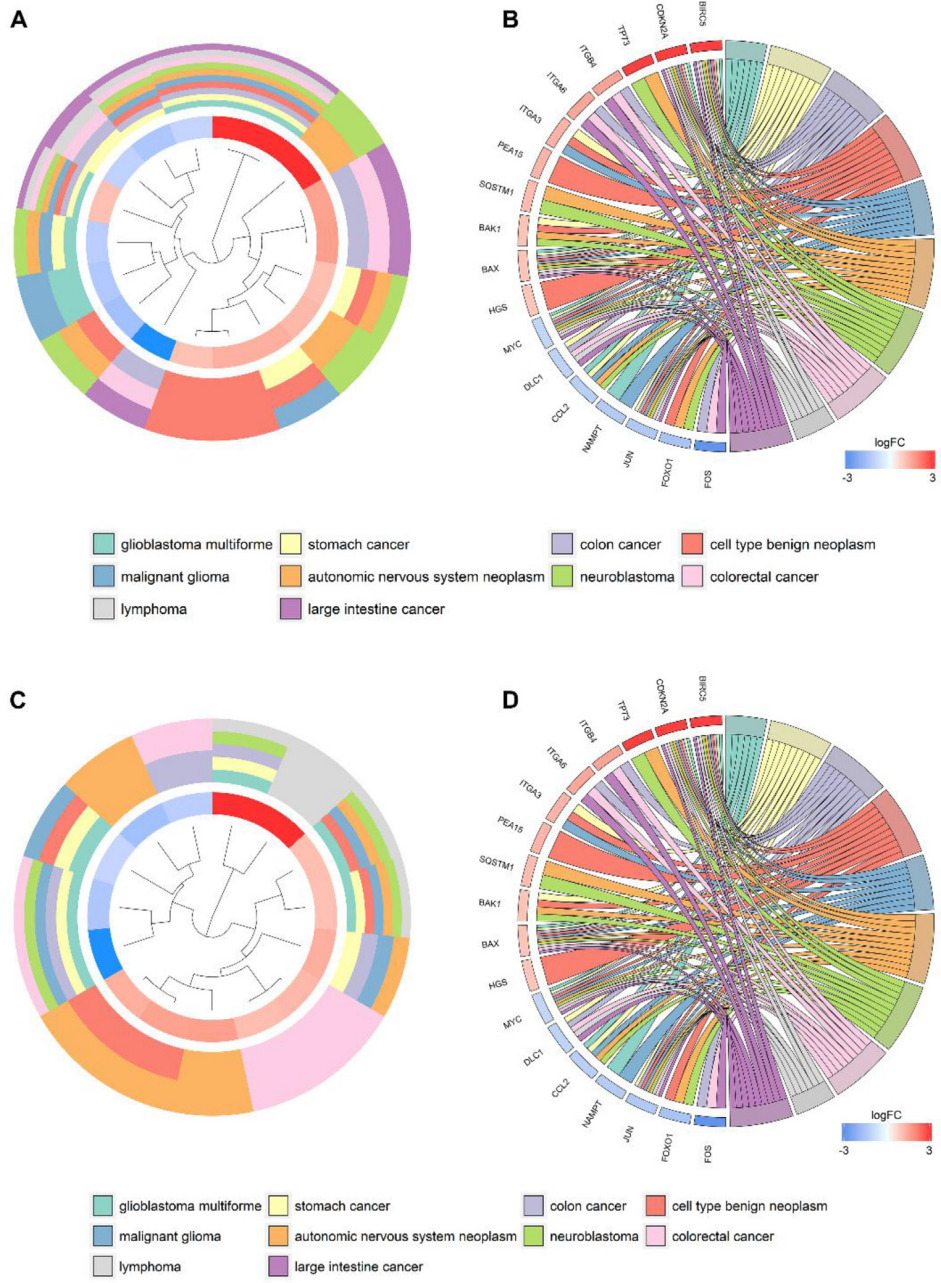
Disease Ontology (DO) and Kyoto Encyclopedia of Genes and Genomes (KEGG) analyses of the differentially expressed genes Circle plot displaying a circular dendrogram of the clustering of the expression profiles. The inner ring shows the color-coded logFC, the outer ring the assigned DO (**A**) and KEGG (**C**) terms. Cluster displaying these genes are linked via ribbons to their assigned DO (**B**) and KEGG (**D**) terms. Blue-to-red coding next to the selected genes indicates logFC.

According to the gene functional annotation summarized by clusterProfiler package of R software, we obtained Gene Ontology (GO), Disease Ontology (DO) and Kyoto Encyclopedia of Genes and Genomes (KEGG) annotations of differently expressed autophagy-related genes in HCC. In the biological process (BP) term of GO analysis, not surprisingly, we found these genes were significantly related to autophagy (Figure 7A; Figure 8A–8B). In cellular component (CC), “autophagosome”, “integrin complex” and “protein complex involved in cell adhesion” were significantly enriched by these genes (Figure 7C; Figure 8C–8D). For molecular function (MF), “sequence-specific binding”, “growth factor binding” and “transcription factor binding” were items for these genes to play important roles (Figure 7E; Figure 8E–8F). Interestingly, DO analysis indicated that these genes were also mainly related to various types of tumors, such as neuroblastoma, colorectal and stomach cancers (Figure 8B; Figure 9A–9B). Do analysis provided us clues that these genes could act as general oncogenes. We also found these genes enriched in several risk KEGG pathways which are relevant to tumor initiation and progression (Figure 7D; Figure 9C–9D).

### Autophagy-related prognosis index and clinicopathological parameters

**Figure 10:**
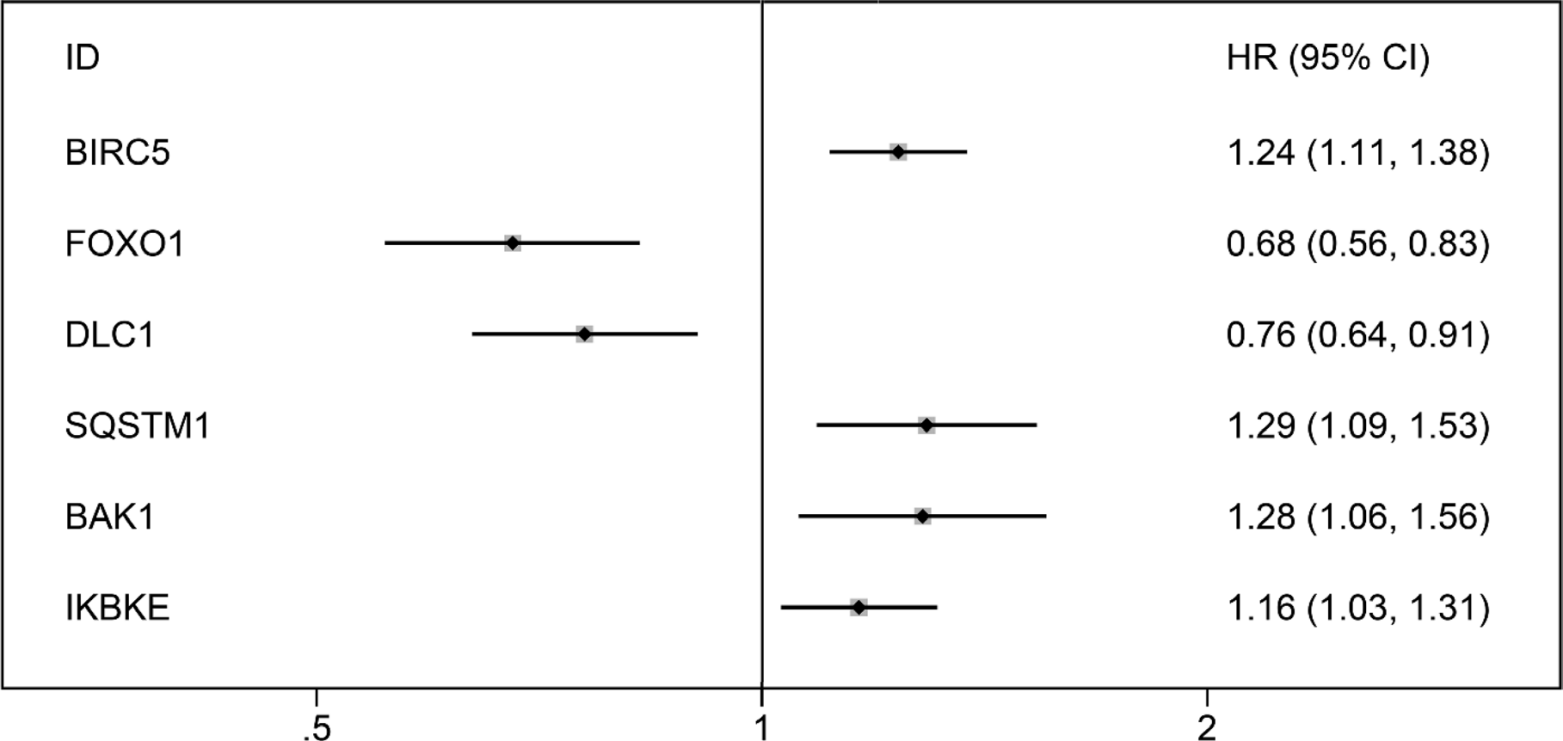
Forrest plots of hazard ratios of survival associated autophagy-related genes in HCC The order is arranged in terms of *P* values.

**Figure 11:**
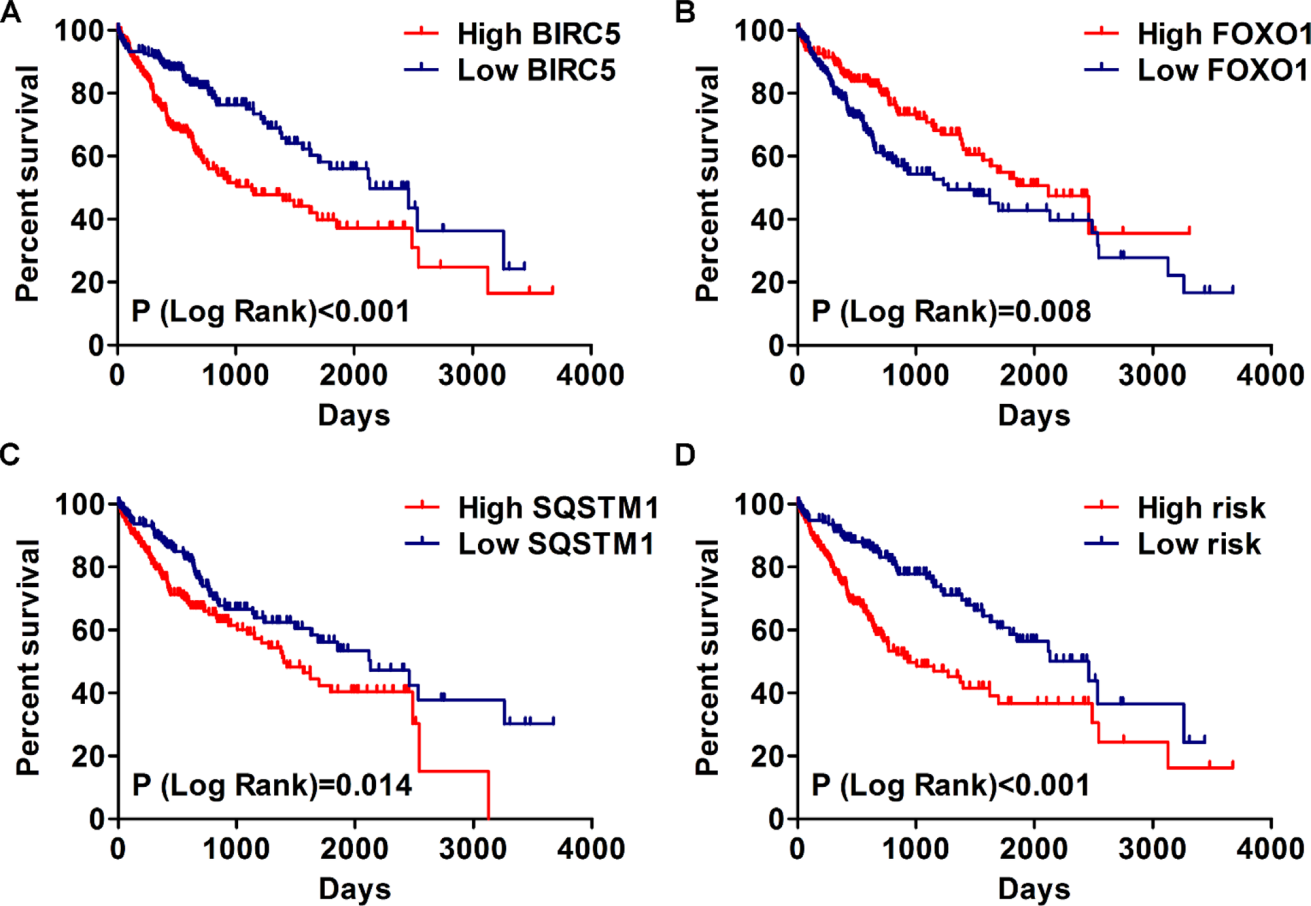
The correlation between prognosis index and HCC patients’ survival (**A**) Kaplan–Meier (K-M) analysis represented that patients in higher BIRC5 group had significantly shorter overall survival (OS) time than those in lower BIRC5 group. (**B**) K-M analysis represented that patients in higher FOXO1 group had significantly longer OS time than those in lower FOXO1 group. (**C**) K-M analysis represented that patients in higher SQSTM1 group had significantly shorter OS time than those in lower SQSTM1 group. (**D**) K-M analysis represented that patients in high-risk group had significantly shorter OS time than those in low-risk group.

After we removed the patients without sufficient survival data, a final cohort of 371 HCC patients in TCGA was used for prognosis evaluation. The univariate Cox analysis revealed that 6 genes including BIRC5, FOXO1, DLC1, SQSTM1, BAK1 and IKBKE had a prognostic value for HCC (Figure 10). Subsequently, multivariate Cox analysis was conducted and BIRC5, FOXO1 and SQSTM1 were screened as independent prognostic indicators for overall survival (OS) of HCC (Figure 11A–11C; Supplementary Table 1).

Furthermore, *t*-test analyses indicated that the three genes were differentially expressed in various clinicopathological parameters. As shown in Table 1, differential BIRC5 expression was found at different tumor status, pathological stages, histological grades, T stages and vascular tumor cell types. Differential expression of FOXO1 was observed in different histological grades and vascular tumor cell types. For SQSTM1, it showed different expression in terms of age, gender and vascular tumor cell type.

**Table 1:** Relationships between three genes expression and clinical parameters in TCGA

Parameters		N	BIRC5 expression value			FOXO1 expression value			SQSTM1 expression value		
			M ± SD	*t* value	*P* value	M ± SD	*t* value	*P* value	M ± SD	*t* value	*P* value
Tissues	HCC	374	9.4369 ± 1.6201	21.454	<0.001	9.9104 ± 0.9773	–12.320	<0.001	14.6186 ± 1.0846	9.352	<0.001
	Non-tumor	50	5.5929 ± 1.1200			11.6892 ± 0.8051			13.8585 ± 0.4160		
Age	≥60	201	9.3100 ± 1.5752	–1.441	0.150	9.9202 ± 0.9826	0.349	0.727	14.7339 ± 1.1063	2.218	0.027
	<60	169	9.4965 ± 0.7752			9.8846 ± 0.9731			14.4841 ± 1.0454		
Gender	Male	250	9.3990 ± 1.6469	–0.430	0.667	9.9343 ± 0.9854	0.844	0.399	14.7864 ± 1.1160	4.249	<0.001
	Female	121	9.4762 ± 1.5659			9.8430 ± 0.9571			14.2869 ± 0.9379		
Tumor status	With tumor	151	9.6837 ± 1.5749	2.731	0.007	9.8336 ± 0.9601	–1.234	0.218	14.6803 ± 1.1622	1.051	0.294
	Tumor free	201	9.2128 ± 1.6205			9.9643 ± 1.0012			14.5567 ± 1.0378		
Histological grade	G3∼G4	134	10.0538 ± 1.3480	6.165	<0.001	9.6820 ± 0.9752	–3.350	0.001	14.7429 ± 1.2175	1.395	0.164
	G1∼G2	232	9.0719 ± 1.6550			10.0340 ± 0.9643			14.5700 ± 0.9988		
Pathologic stage	III∼IV	90	9.8116 ± 1.7962	2.261	0.025	9.7568 ± 0.8773	–1.719	0.086	14.5065 ± 1.1635	–1.053	0.293
	I∼II	257	9.3339 ± 1.5039			9.9646 ± 1.0221			14.6466 ± 1.0582		
T stage	T3–T4	93	9.8389 ± 1.7861	2.276	0.006	9.7376 ± 0.8663	–1.892	0.059	14.5563 ± 1.1834	–0.753	0.452
	T1–T2	275	9.3106 ± 1.5225			9.9592 ± 1.0104			14.6545 ± 1.0534		
N stage	N1-3	4	8.9554 ± 2.3328	–0.707	0.480	9.8236 ± 0.8663	–0.111	0.911	13.5898 ± 1.3395	–1.873	0.062
	N0	252	9.5184 ± 1.5693			9.8771 ± 0.9552			14.6407 ± 1.1101		
M stage	M1	4	9.5272 ± 0.4724	–0.058	0.957	9.5522 ± 0.4742	–0.623	0.534	14.6652 ± 0.4200	0.063	0.950
	M0	266	9.5421 ± 1.6475			9.8602 ± 0.9853			14.6295 ± 1.1343		
Vascular tumor cell type	Micro/Macro	109	9.5617 ± 1.6224	1.995	0.047	9.8887 ± 0.9702	–1.210	0.227	14.7845 ± 1.1732	2.051	0.045
	None	205	9.1905 ± 1.5410			10.0240 ± 0.9290			14.5311 ± 0.9965		

M ± SD: Mean ± Std. Deviation.

**Figure 12:**
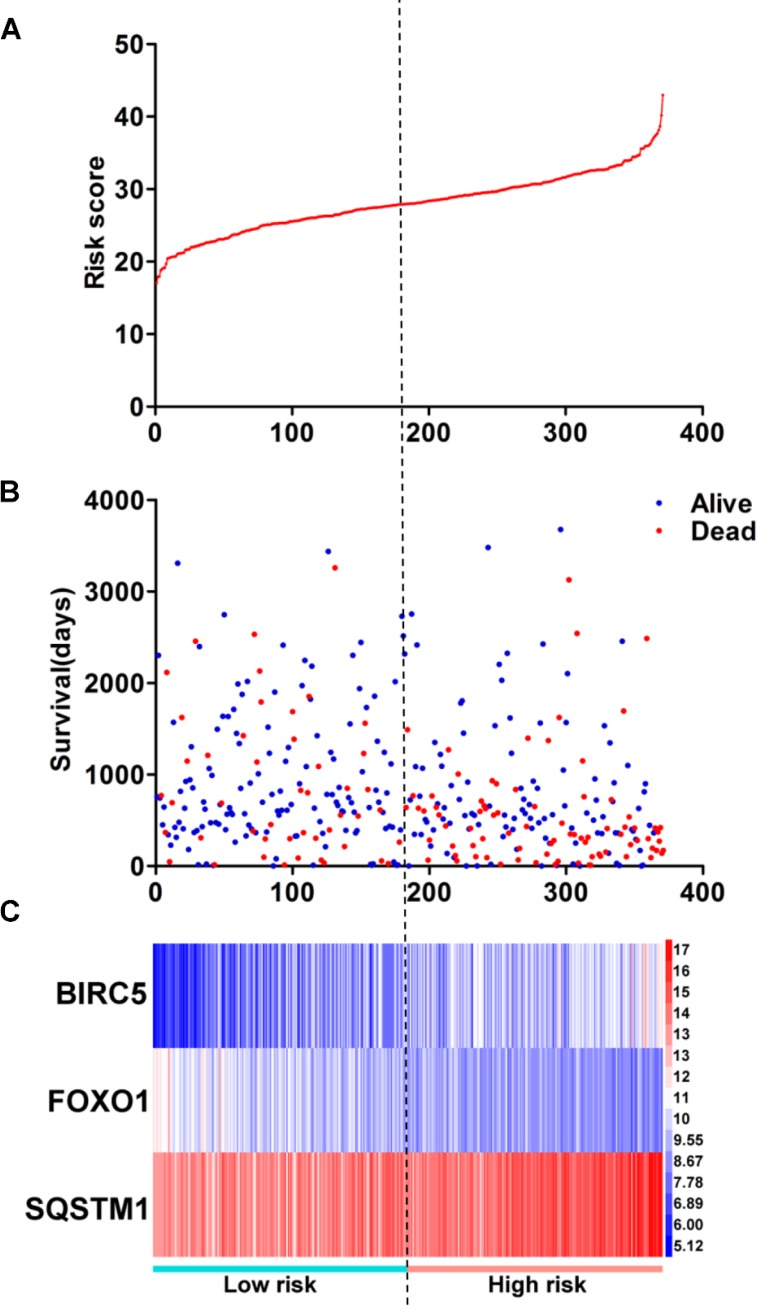
Prognosis index for HCC patients (**A**) The low and high score group for the prognosis index in HCC patients; (**B**) The survival status and duration of HCC cases; (**C**) Heatmap of the three genes expression in HCC. The color from blue to red shows a trend from low expression to high expression.

**Figure 13:**
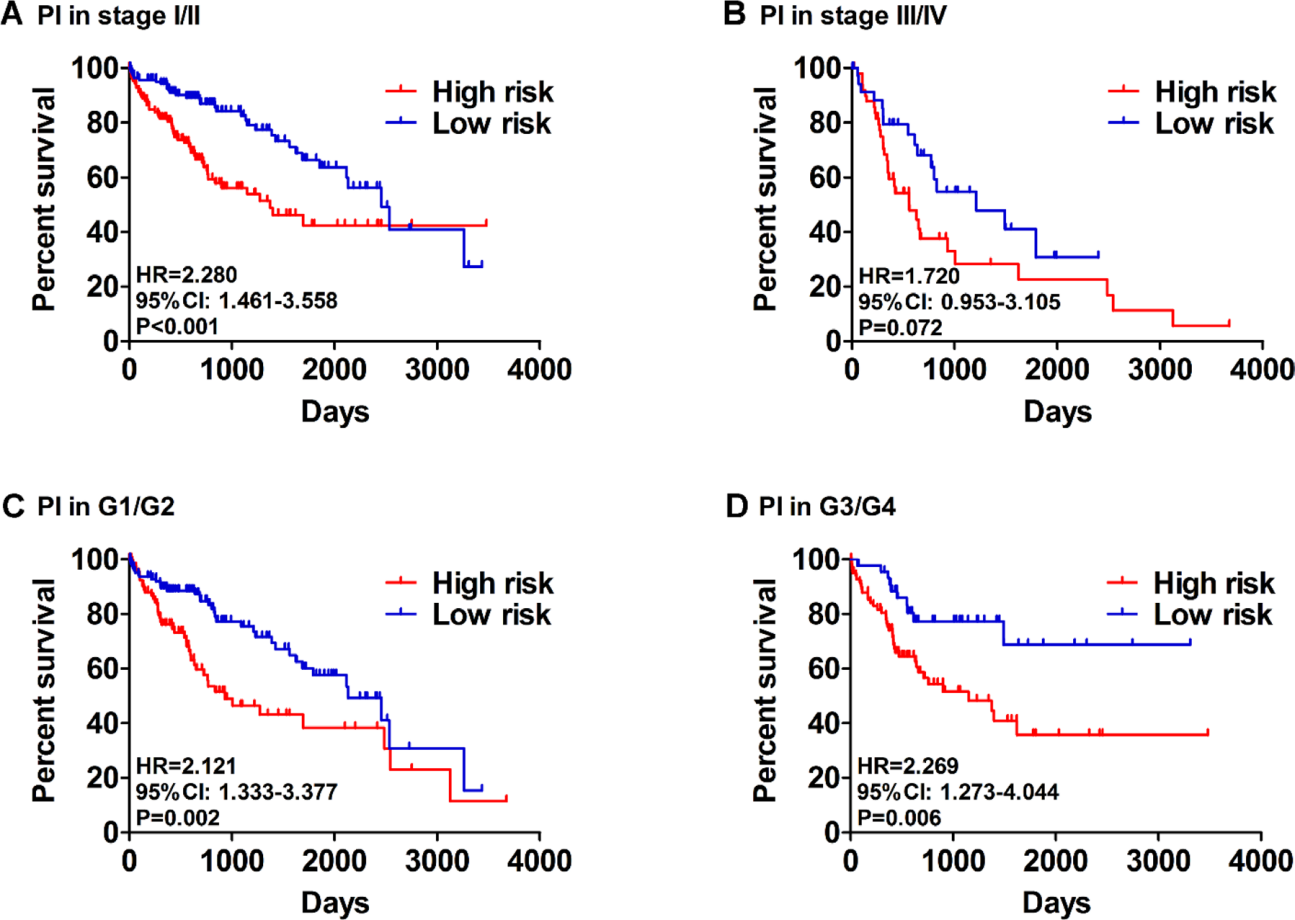
Patients were stratified by prognosis index (high- and low- risk) (**A**) Overall survival (OS) among HCC patients with stage I and II. (**B**) OS among HCC patients with stage III and IV. (**C**) OS among HCC patients with stage I and II. (**D**) OS among HCC patients with stage I and II.

**Figure 14:**
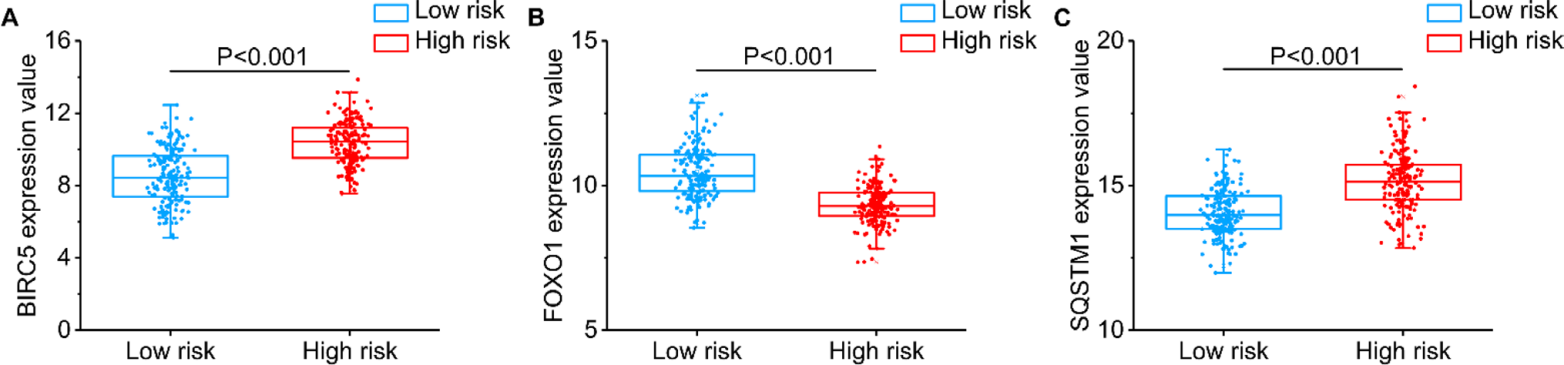
Different expression of the three genes between high risk group and low risk group (**A**) BIRC5, (**B**) FOXO1, (**C**) SQSTM1.

Then, we generated a risk score model PI for predicting OS based on the three genes (Figure 12) using the formula: PI = (1.242 * expression level of BIRC5) + (–1.619 * expression level of FOXO1) + (2.214 * expression level of SQSTM1). Then, the PI significantly stratified each HCC patients into high-risk (*n* = 186) or low-risk (*n* = 185) groups in terms of the OS (HR = 2.179, 95% CI: 1.537–3.089, *P <* 0.001; Figure 11D). Multivariate analysis demonstrated a consistent HR of 1.930 (95% CI: 1.200–3.104, *P* = 0.007, Table 2), which verified that the PI composed of the three genes remained as an independent prognostic indicator for the OS of HCC patients. When the HCC patients were stratified by pathological stage, the PI remained a high prognostic value for stage I/II (HR = = 2.280, 95CI%:1.461–3.558, *P <* 0.001; Figure 13A) and stage III/IV (HR = 1.720, 95CI%:0.953–3.105, *P* = 0.072; Figure 13B). When restricted to HCC patients with histological grades, a higher PI was still relevant with patients in G1/G2 (HR = 2.121, 95CI%:1.333–3.377, *P* = 0.002; Figure 13C) and G3/G4 (HR = 2.269, 95CI%:1.273–4.044, *P* = 0.006; Figure 13D). We also assessed the expression pattern of the three genes between the high–and low-risk groups. The results of data analysis showed that a remarkably higher expression for BIRC5 and SQSTM1 in the high-risk groups, while they had lower expression for FOXO1 in the high-risk groups (Figure 14).

**Table 2:** Univariate and multivariate analyses of OS in HCC patients of TCGA

Variables	Univariate analysis		Multivariate analysis	
	Hazard ratio (95% CI)	*P* value	Hazard ratio (95%CI)	*P* value
Age (≥60/<60)	1.212 (0.854–1.720)	0.281		
Gender (male/female)	0.817 (0.573–1.164)	0.262		
Pathologic stage (III–IV/I–II)	2.446 (1.687–3.545)	<0.001	1.492 (0.203–10.938)	0.694
Tumor (T3–T4/T1–T2)	2.537 (1.783–3.609)	<0.001	1.517 (0.206–11.164)	0.682
Lymph node metastasis(yes/no)	1.999 (0.490–8.161)	0.334		
Distant metastasis (yes/no)	4.033 (1.267–12.834)	0.018	1.019 (0.241–4.313)	0.979
Histologic grade (G3–G4/G1–G2)	1.119 (0.780–1.604)	0.542		
Tumor status (with tumor/tumor free)	2.366 (1.623–3.447)	<0.001	1.972 (1.236–3.148)	0.004
Vascular tumor cell type (Micro+Macro/None)	1.351 (0.892–2.047)	0.155		
PI (High risk/Low risk)	2.175 (1.522–3.108)	<0.001	1.930 (1.200–3.104)	0.007

PI: prognosis index.

### Validation of PI as an effective prognostic factor

**Figure 15:**
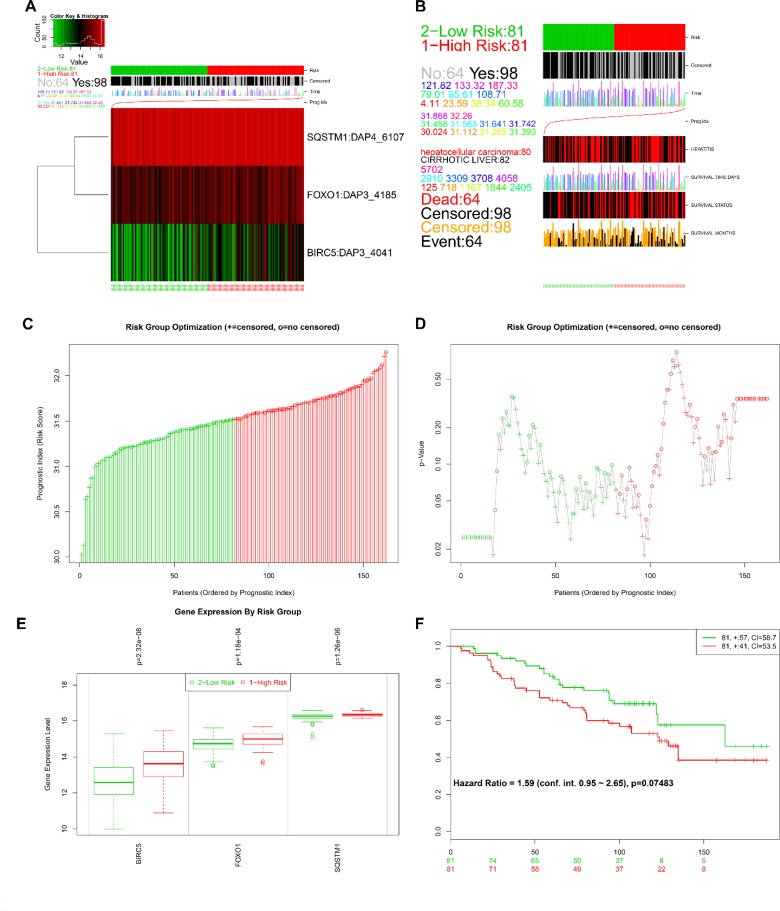
Evaluation of prognosis index in the survival of HCC patients in GSE10143 database (**A**) A heatmap representation of the gene expression values. (**B**) Clinical information available related to risk group, prognosis index (PI) and clinical outcome. (**C**) The low and high score group for the PI in HCC patients. (**D**) A box plot across risk groups ordered by PI. (**E**) Different expression of the three genes between high risk group and low risk group. (**F**) Kaplan-Meier analysis represented that patients in high-risk group had shorter overall time than those in low-risk group.

**Figure 16:**
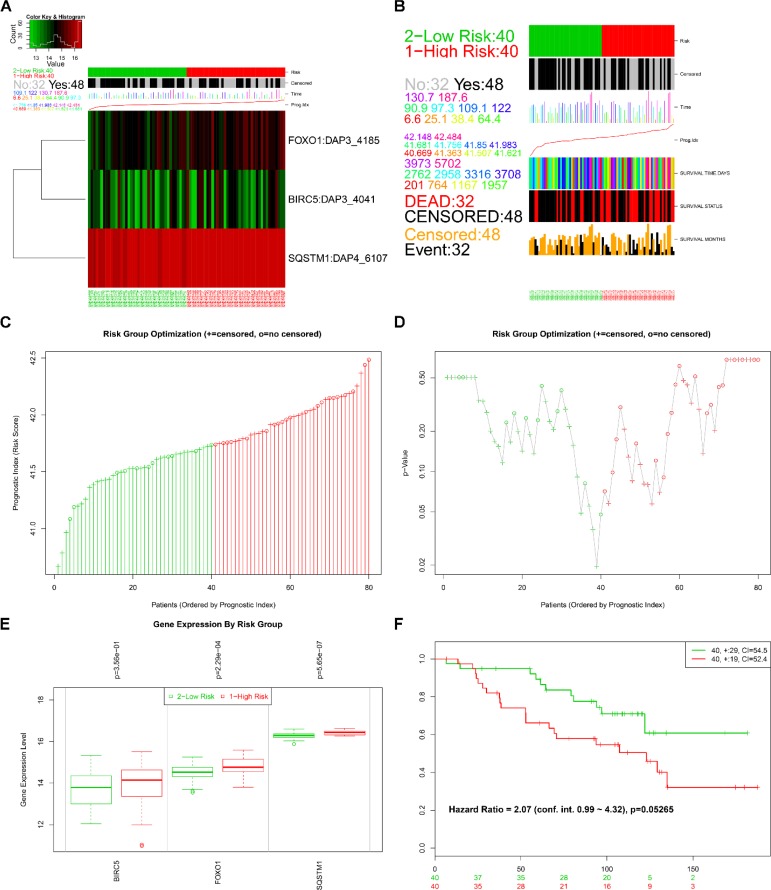
Evaluation of prognosis index in the survival of HCC patients in GSE10186 database (**A**) A heat map representation of the gene expression values. (**B**) Clinical information available related to risk group, prognosis index (PI) and clinical outcome. (**C**) The low and high score group for the PI in HCC patients. (**D**) A box plot across risk groups ordered by PI. (**E**) Different expression of the three genes between high risk group and low risk group. (**F**) Kaplan-Meier analysis represented that patients in high-risk group had shorter overall time than those in low-risk group.

**Figure 17:**
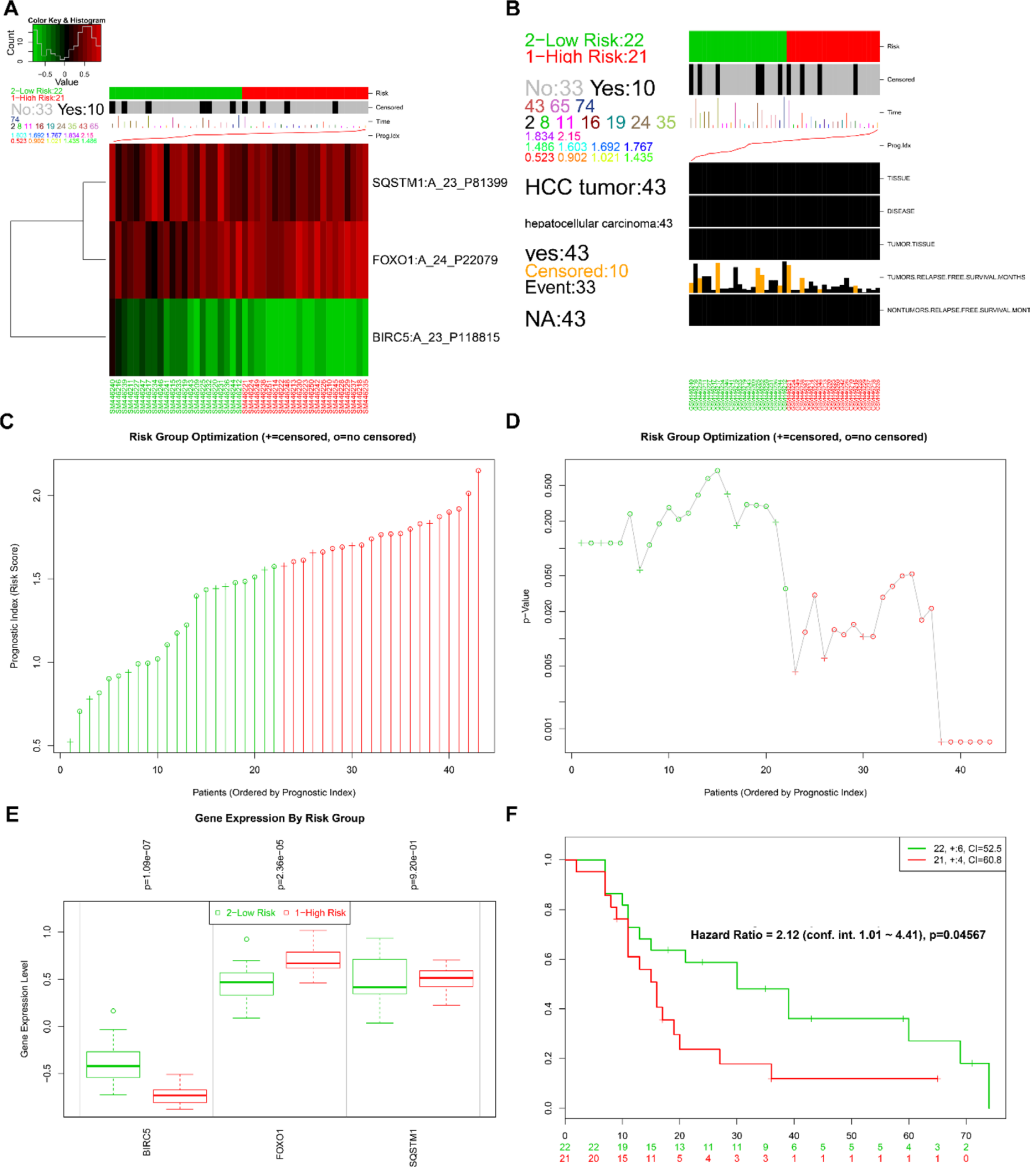
Evaluation of prognosis index in the survival of HCC patients in GSE17856 database (**A**) A heat map representation of the gene expression values. (**B**) Clinical information available related to risk group, prognosis index (PI) and clinical outcome. (**C**) The low and high score group for the PI in HCC patients. (**D**) A box plot across risk groups ordered by PI. (**E**) Different expression of the three genes between high risk group and low risk group. (**F**) Kaplan-Meier analysis represented that patients in high-risk group had shorter relapse free survival time than those in low-risk group.

The online bioinformatics tool SurvExpress provides the differences in mRNA expression levels to draw Kaplan–Meier curves and to classify risk groups. Groups with low and high expression are represented by green and red, respectively. SurvExpress contained two datasets with OS data (GSE10143, *N* = 162 and GSE10186, *N* = 112) and 1 dataset with recurrence free survival (RFS; GSE17856, *N* = 95) data. We first exhibited the expression pattern of these 3 genes (Figure 15A, 16A, 17A) and clinical information of patients included in datasets (Figure 15B, 16B, 17B). And then, similar to from the strategy of TCGA data dealing, patients in the three datasets from GEO were stratified into high- or low-risk groups by PI signature (Figures 15C–15D, 16C–16D, 17C–17D). For GSE10143 cohort, patients in high-risk group had inferior OS than patients in low-risk group (HR = 1.95, 95% CI = 0.95–2.65, *P* = 0.075; Figure 15E–15F). For GSE10186 cohort, patients in high-risk group also had inferior OS than patients in low-risk group (HR = 2.07, 95% CI = 0.99–4.32, *P* = 0.053; Figure 16E–16F). In the GSE17856 cohort, PI could function as an indicator for the RFS of HCC patients. (HR = 2.12, 95% CI = 1.01–4.41, *P =* 0.046; Figure 17E–17F). These findings provided a valuable message that combination of these three genes could act as an indicator of prognosis monitoring with moderate degree.

### Expression profiling of identified prognostic genes

**Figure 18:**
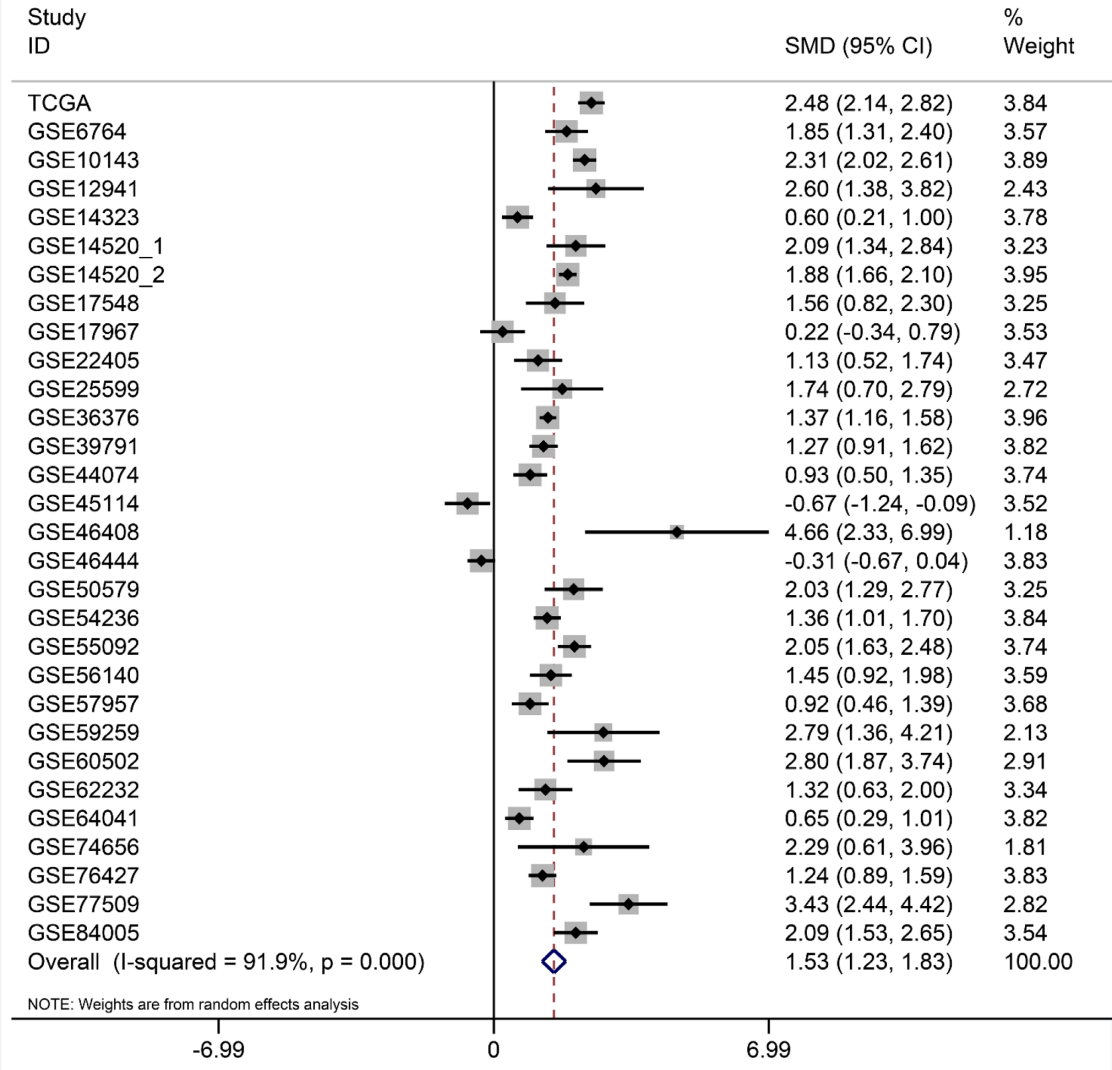
Forest plots for meta-analysis of evaluating BIRC5 expression between HCC and non-tumor tissues SMD and associated 95% confidence interval were calculated using the random effects model.

**Figure 19:**
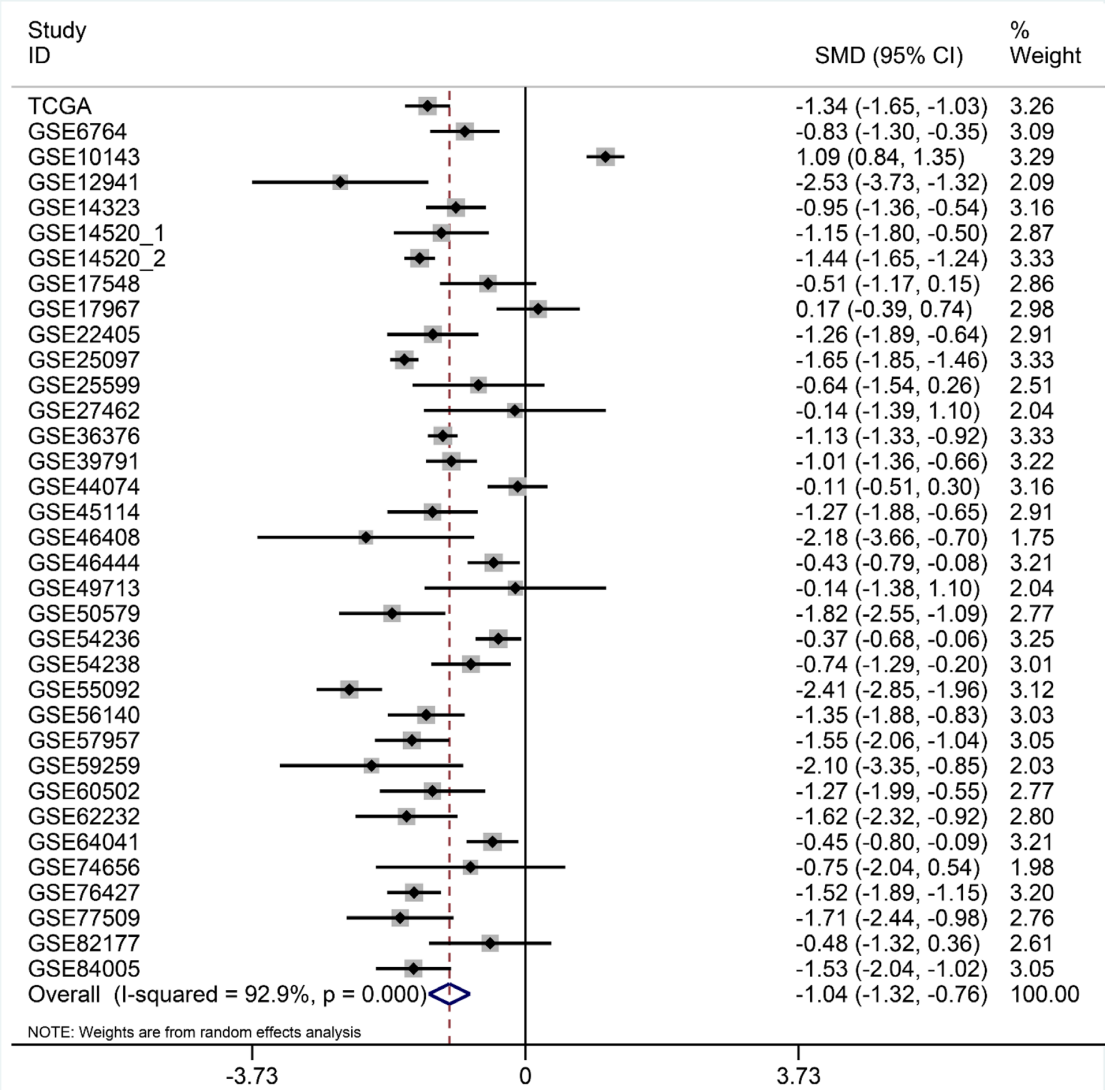
Forest plots for meta-analysis of evaluating SQSTM1 expression between HCC and non-tumor tissues SMD and associated 95% confidence interval were calculated using the random effects model.

**Figure 20:**
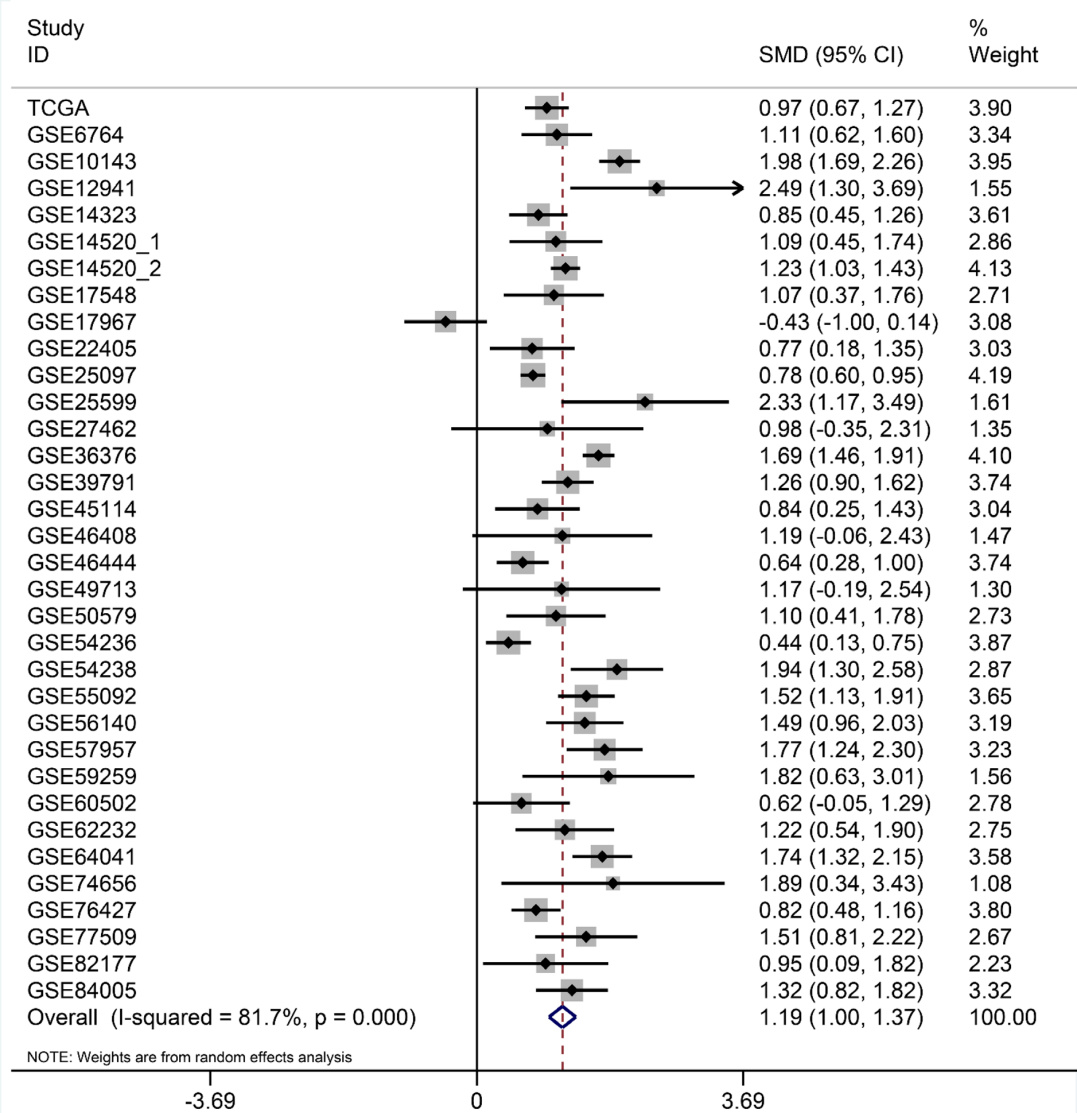
Forest plots for meta-analysis of evaluating SQSTM1 expression between HCC and non-tumor tissues SMD and associated 95% confidence interval were calculated using the random effects model.

**Figure 21:**
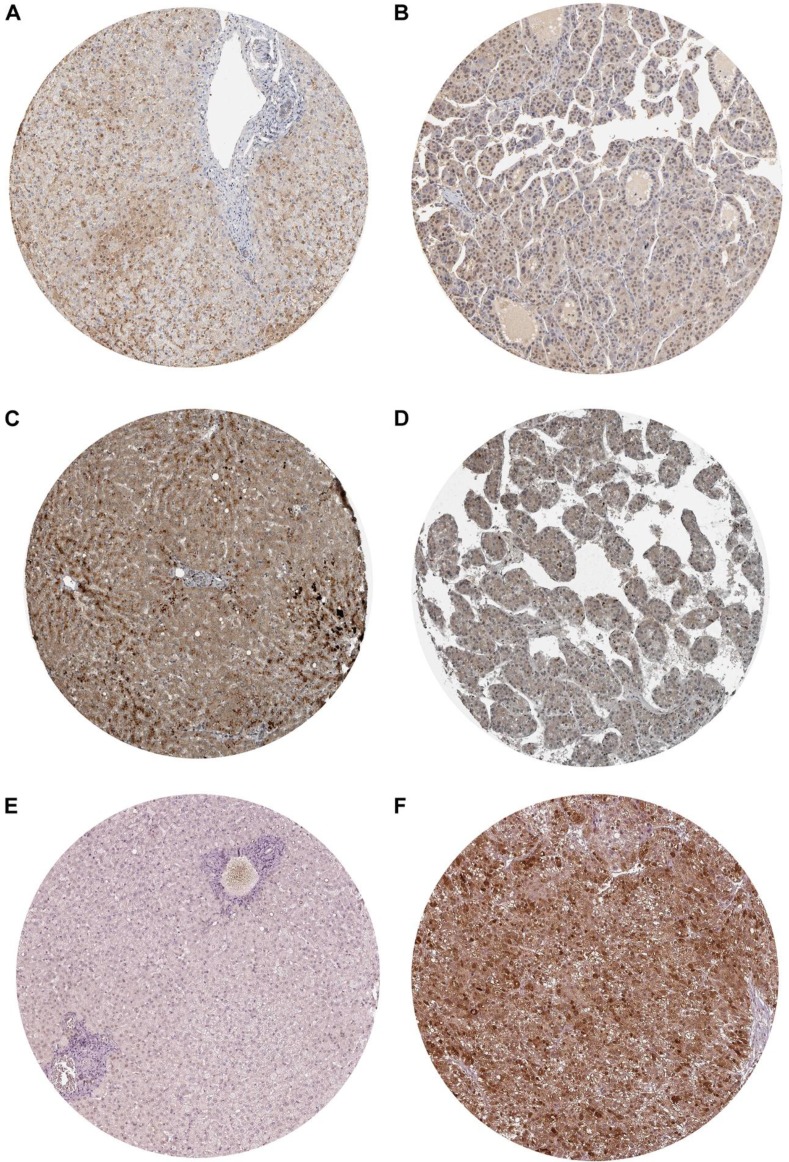
Validation of the protein expression of BIRC5, FOXO1 and SQSTM1 in HCC by Immunohistochemistry based on proteinatlas database (**A**) IHC staining of BIRC5 in normal liver Magnification: 200um; Antibody: HPA002830. (**B**) IHC staining of BIRC5 in hepatocellular carcinoma. Magnification: 200um; Antibody: HPA002830. (**C**) IHC staining of FOXO1 in normal liver. Magnification: 200um; Antibody: CAB022326. (**D**) IHC staining of FOXO1 in hepatocellular carcinoma. Magnification: 200um; Antibody: CAB022326. (**E**) IHC staining of SQSTM1 in normal liver. Magnification: 200um; Antibody: CAB004587. (**F**) IHC staining of SQSTM1 in hepatocellular carcinoma. Magnification: 200um; Antibody: CAB004587.

After searching and selecting HCC-related datasets in the GEO, a total of 34 GEO datasets were used to identify the expression pattern of BIRC5, FOXO1 and SQSTM1. The characteristics of included datasets were shown in Table 3. After integrating the TCGA data and these datasets by using Standardized Mean Difference (SMD), we found that the BIRC5 expression level was significantly increased in the tumor group (SMD = 1.52, 95% CI: 1.23–1.83, *P* < 0.001; Figure 18) when using the random-effects model. And FOXO1 was validated to be lower in HCC (SMD = –1.04, 95% CI: –1.32–(–0.76), *P* < 0.001; Figure 19) when using the random-effects model. For SQSTM1, its expression level was significantly upregulated in tumor group (SMD = 1.19, 95% CI: 1.00–1.37, *P <* 0.001; Figure 20). Moreover, remarkable overexpression of BIRC5 and SQSTM1 and opposite expression of FOXO1 protein in HCC was confirmed by Proteinatlas (Figure 21).

**Table 3:** General characteristics of included datasets

Dataset	First author	Publication year	Country	Sample source	Data source	Platform	Number of HCC samples	Number of non-tumor samples
GSE6764	Wurmbach E *et al.*	2007	USA	Tissue	GEO	Affymetrix GPL570	35	40
GSE10143	Hoshida Y *et al.*	2008	USA	Tissue	GEO	Illumina GPL5474	80	307
GSE12941	Yamada T *et al.*	2010	Japan	Tissue	GEO	Affymetrix GPL5175	10	10
GSE14321	Mas VR *et al.*	2009	USA	Tissue	GEO	Affymetrix GPL571	38	77
GSE14520_1	Roessler S *et al.*	2010	USA	Tissue	GEO	Affymetrix GPL571	22	21
GSE14520_2	Roessler S *et al.*	2010	USA	Tissue	GEO	Affymetrix GPL3921	225	220
GSE17548	Ozturk M *et al.*	2013	Turkey	Tissue	GEO	Affymetrix GPL570	17	20
GSE17967	Archer KJ *et al.*	2009	USA	Tissue	GEO	Affymetrix GPL571	16	47
GSE22405	Zhang HH *et al.*	2014	USA	Tissue	GEO	Affymetrix GPL10553	24	24
GSE25097	Zhang C *et al.*	2011	USA	Tissue	GEO	Rosetta GPL10687	268	289
GSE25599	Xing J *et al.*	2013	China	Tissue	GEO	Illumina GPL9052	10	10
GSE27462	Yang F *et al.*	2011	China	Tissue	GEO	Arraystar GPL11269	5	5
GSE36376	Lim HY *et al.*	2012	South Korea	Tissue	GEO	Illumina GPL10558	240	193
GSE39791	Kim J et al.	2014	USA	Tissue	GEO	Illumina GPL10558	72	72
GSE44074	Ueda T *et al.*	2013	Japan	Tissue	GEO	Kanazawa GPL13536	34	71
GSE45114	Wei L *et al.*	2013	China	Tissue	GEO	CapitalBio GPL5918	24	25
GSE46408	Jeng Y *et al.*	2013	Taiwan	Tissue	GEO	Agilent GPL4133	6	6
GSE46444	Chen X *et al.*	2014	USA	Tissue	GEO	Illumina GPL13369	88	48
GSE49713	Wang K *et al.*.	2013	China	Tissue	GEO	Arraystar GPL11269	5	5
GSE50579	Geffers R *et al.*	2013	Germany	Tissue	GEO	Agilent GPL14550	67	10
GSE54236	Villa E *et al.*	2014	Italy	Tissue	GEO	Agilent GPL6480	81	80
GSE54238	Yuan S *et al.*	2014	USA	Tissue	GEO	Arraystar GPL16955	26	30
GSE55092	Melis M *et al.*	2014	USA	Tissue	GEO	Affymetrix GPL570	49	91
GSE56140	Hoshida Y *et al.*	2014	USA	Tissue	GEO	Illumina GPL18461	35	34
GSE57957	Mah W *et al.*	2014	Singapore	Tissue	GEO	Illumina GPL10558	39	39
GSE59259	Udali S *et al.*	2015	Italy	Tissue	GEO	NimbleGen PL18451	8	8
GSE60502	Kao KJ *et al.*	2015	Taiwan	Tissue	GEO	Affymetrix GPL96	18	18
GSE62232	Zucman-Rossi J *et al.*	2014	France	Tissue	GEO	Affymetrix GPL570	81	10
GSE64041	Makowska Z *et al.*	2016	Switzerland	Tissue	GEO	Affymetrix GPL6244	60	65
GSE74656	Tao Y *et al.*	2015	China	Tissue	GEO	Affymetrix GPL16043	5	5
GSE76427	Grinchuk OV *et al.*	2017	Singapore	Tissue	GEO	Illumina GPL10558	115	52
GSE77509	Jin G *et al.*	2017	China	Tissue	GEO	Illumina GPL16791	20	20
GSE82177	Wijetunga NA *et al.*	2016	USA	Tissue	GEO	Illumina GPL11154	8	19
GSE84005	Tu X *et al.*	2017	China	Tissue	GEO	Affymetrix PL5175	38	38

### Identification of clinical significances by in-house immunohistochemistry

**Figure 22:**
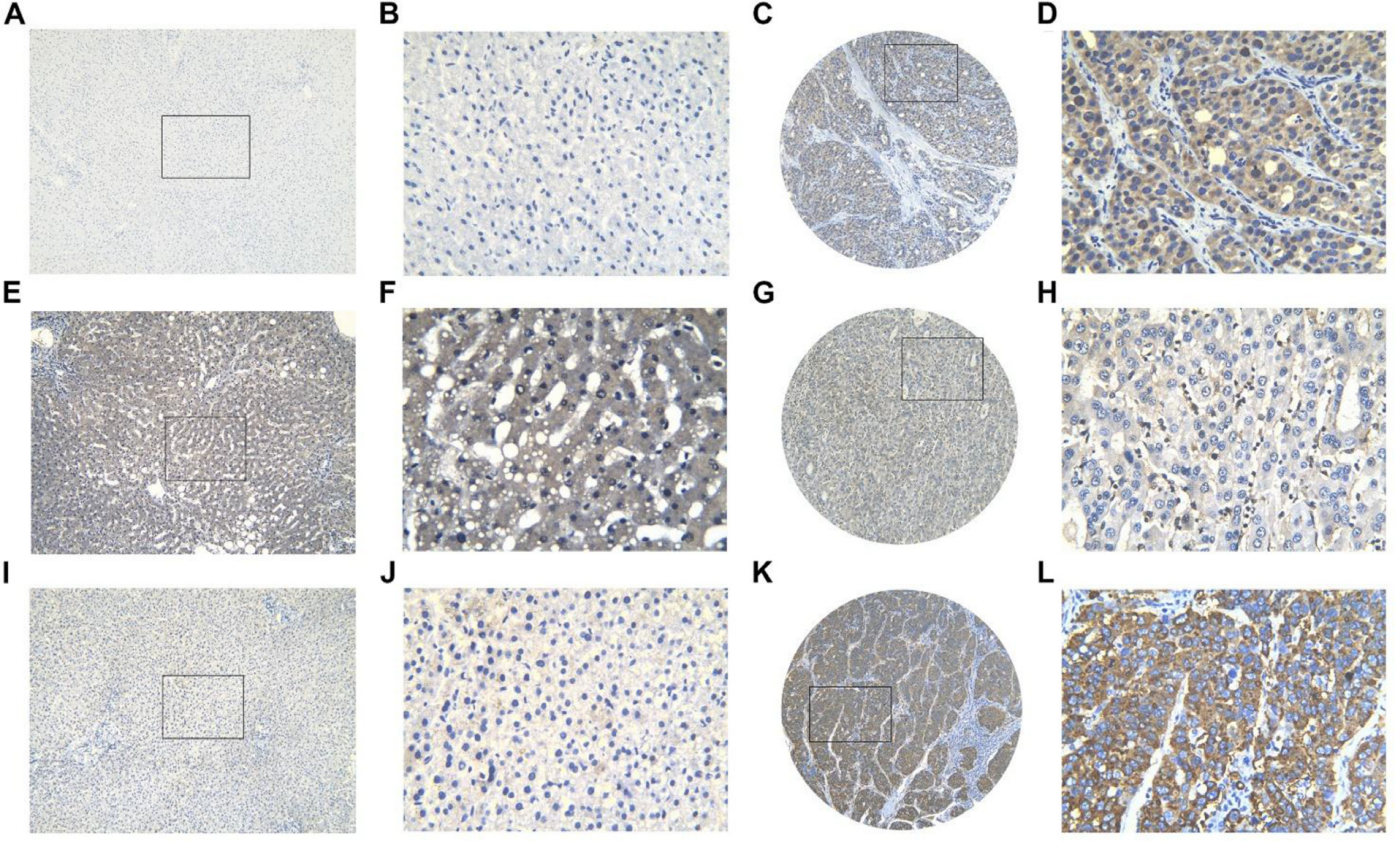
Three protein expression in HCC and non-tumor tissues assessed by immunohistochemistry BIRC5 showed low expression in non-tumor tissue (**A**, **B**) and high expression in HCC tissue (**C**, **D**); FOXO1 showed high expression in non-tumor tissue (**E**, **F**) and low expression in HCC tissue (**G**, **H**); SQSTM1 showed low expression in non-tumor tissue (**I**, **J**) and high expression in HCC tissue (K, L). Magnification: ×100 (A, C, E, G, I, K) or ×400 (B, D, F, H, J, L).

BIRC5, FOXO1 and SQSTM1 protein expression level were detected by immunohistochemistry (IHC) in 302 HCC tissues and in 41 non-tumor tissues. For BIRC5, 97 samples (32.12%) were assigned to the positive group and 205 cases (67.88%) were assigned to the negative group according to the immunoreactive score (IRS) of the tissue samples (Figure 22C–22D). In non-tumor tissues, 2 samples (4.9%) were exhibited positive BIRC5 expression, and 39 (95.12%) were negative (Figure 22A–22B). HCC and non-tumor tissues exerted statistical difference in BIRC5 protein expression (*P <* 0.001). Similarly, the IHC results of SQSTM1 also confirmed its higher expression in HCC tissues. A total of 152 (50.33%) HCC tissues were positive while 150 (49.67%) samples were negative (Figure 22K–22L). The positive rate was significantly higher than non-tumor tissues (*P =* 0.001), which showed low positive rate (9/41, 21.95%) (Figure 22I–22J). The data of IHC analysis demonstrated that positive FOXO1 expression was detected in 190 of 302 HCC tissues (62.91%) (Figure 22G–22H), whereas 29 of 41 non-tumor tissues (70.73%) showed high FOXO1 expression (Figure 22E–22F). Although there was no significant difference, which may be owed to the limited samples, FOXO1 still showed a high positive rate in non-tumor tissues.

The correlations between these genes expression and clinicopathological features of HCC are presented in Table 4. We observed significant positive correlations between BIRC5 expression and age (*P =* 0.019) and the pathologic T stage (*P =* 0.017). Up-regulated SQSTM1 also showed notable relationship with advanced pathologic T stage (*P =* 0.029). And lower FOXO1 expression was significantly related to advanced TNM stage (*P =* 0.002).

**Table 4:** The relationships between prognostic genes expression and clinicopathological features in HCC in-house

Clinicopathological features	*n*	BIRC5 expression		Chi-squared value	*P*-value	FOXO1 expression		Chi-squared value	*P*-value	SQSTM1 expression		Chi-squared value	*P*-value
		Negative	Positive			Negative	Positive			Negative	Positive		
Tissues													
HCC	302	205	97	13.047	0.001	112	190	0.956	0.328	150	152	11.674	0.001
Non-tumor	41	39	2			12	29			32	9		
Age													
<60	247	175	72	5.485	0.019	89	158	1.111	0.292	121	126	0.252	0.616
≥60	55	30	25			24	31			29	26		
Gender													
Male	256	174	82	0.006	0.938	85	171	0.246	0.620	123	133	1.769	0.184
Female	46	31	15			17	29			27	19		
Pathologic stage													
I–II	252	167	85	1.812	0.178	84	168	9.187	0.002	126	126	0.067	0.796
III–IV	50	38	12			28	22			24	26		
T													
T1–T2	191	139	52	5.709	0.017	78	113	3.257	0.071	104	87	4.752	0.029
T3–T4	111	66	45			34	77			46	65		
N													
N0	276	185	91	0.845	0.358	106	170	2.036	0.153	141	135	2.087	0.149
N1-N3	25	19	6			6	19			9	16		
Metastasis													
M0	299	203	96	-	1.000	111	188	-	0.533	149	150	-	0.498
M1	2	2	0			0	2			0	2		

### Molecular pathways disturbed between high- and low-risk groups

**Figure 23:**
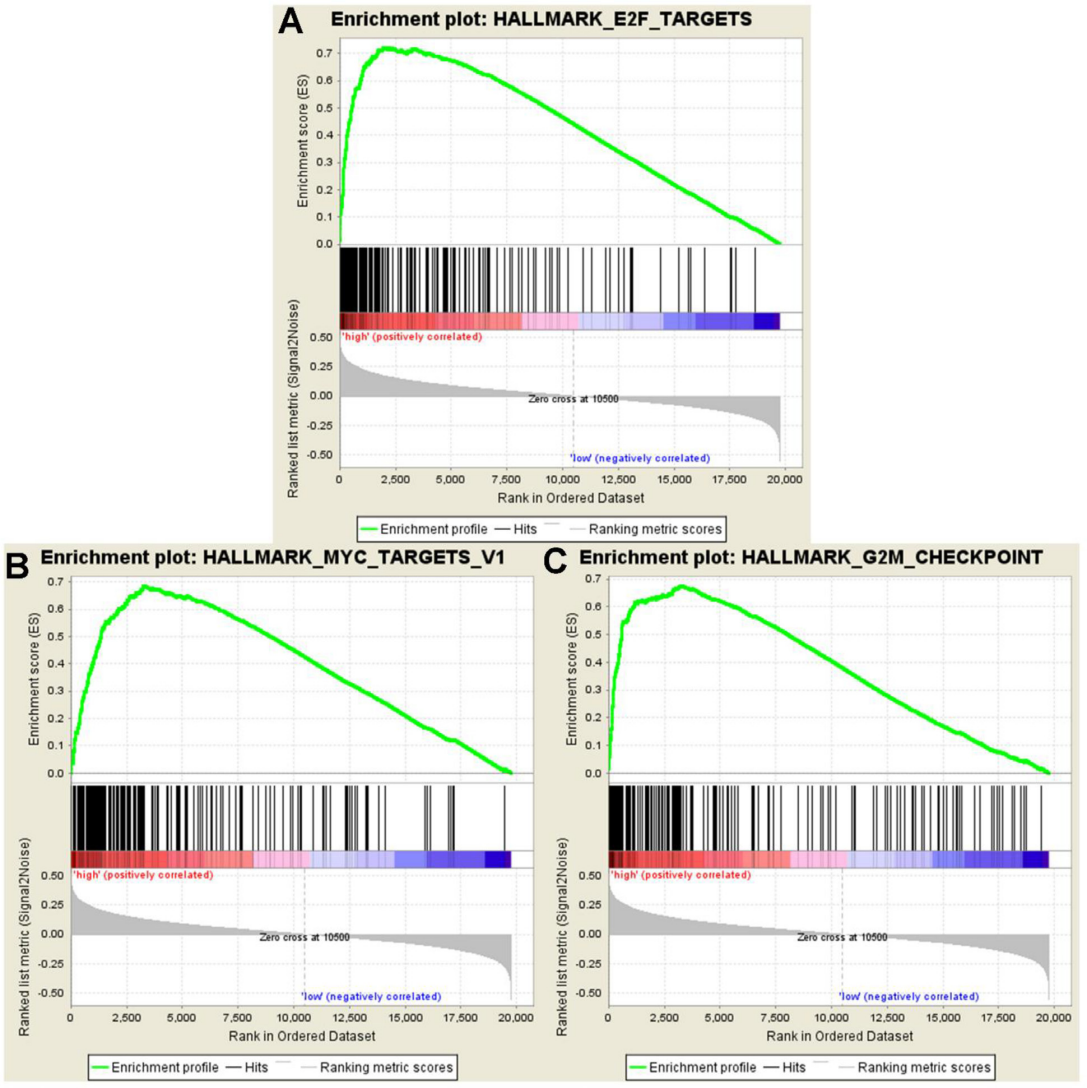
GSEA showed significant enrichment hallmarks in high-risk versus low-risk group (**A**) E2F targets, (**B**) MYC targets, and (**C**) G2M checkpoints.

Gene Set Enrichment Analysis was computed to pick up the molecular pathways disturbed between the high- and low-risk groups. According to the results of the GSEA analysis, genes co-expressed in the high-risk group were significantly enriched in the biological pathways associated with the E2F targets, MYC targets and G2M checkpoint (Figure 23).

## DISCUSSION

HCC patients are at risk for frequently returning cancer and high mortality, even after complete surgical debulking. Reliable molecular biomarkers for HCC prognosis prediction are hence significant for selecting patients who might be sensitive to additional targeted therapy. Using RNA-Seq technology, several novel prognostic indicators based on gene expression profiles have been proposed. Nevertheless, most studies only focused on a signal biomarker, which may not yield sufficient results that are inherently unpredictable and risky. In the present study, we constructed a novel prognostic model based on the expression levels of autophagy-related genes. Furthermore, the prognostic signature was effectively validated against multiple distinct datasets.

In recent years, elaborate molecular research for autophagy in tumors has proposed profound changes in our understanding of tumor management. Katheder NS *et al.* [21] discovered that malignant tumor cells induced autophagy of the surrounding normal cells in the tumor microenvironment and then release amino acids that are absorbed to support tumor growth. The precise mechanism of autophagy in HCC is elusive and changes frequently [22]. Unbalanced autophagy could disturb various vital signaling pathways such as PI3K/AKT/mTOR [23–25], ERK/MAPK [26, 27] and the Wnt/β-catenin [28, 29] and therefore act as an accomplice of malignant hepatocyte to facilitate the HCC progression. In our study, we first identified differentially expressed autophagy-related genes in HCC. Subsequent several gene functional enrichment analyses were performed to assess biological process, disease or pathways influenced by these genes. GO functional enrichment analysis indicated that differentially expressed autophagy-related genes were enriched in autophagy and several proliferation-related biological processes in HCC. KEGG and DO analyzed revealed that dysregulated autophagy-related genes may also play a critical role in the various cancers, which is coincided with other previous studies. These findings suggested that autophagy could be a driver in the process of onset and proliferation of HCC.

Through univariate and multivariate survival analysis, we identified that BIRC5, FOXO1 and SQSTM1 were significantly related with the HCC patients’ overall survival. BIRC5, a member of the anti-apoptosis family, mainly exerted its influence on HCC cells in inhibiting apoptosis [30], promoting proliferation [31], enhancing resistance to radiotherapy and chemotherapy [32] and inducing the tumor stromal angiogenesis [33]. It has been reported that BIRC5 is directly correlated with autophagosome formation and contributed to HCC cells survival [34]. FOXO1 is thought to be a tumor suppressor that is down-regulated in HCC to exert its positive role in reversing the epithelial-to-mesenchymal transition program [35]. However, we still know relatively little about the autophagy-related mechanisms of FOXO1 that control the course of tumorigenesis and tumor development. SQSTM1 is autophagy-adaptor gene and accumulates in autophagy-deficient tumors, including HCC. Its aberrant accumulation and phosphorylation have been a concern in HCC proliferation potency [36, 37] and could represent a potential chemotherapeutic approach against HCC [38, 39]. Our results also evidently corroborated that there are sharp upregulations of BIRC5 and SQSTM1 in HCC while FOXO1 was significantly downregulated by an integrated analytic approach. We then developed a PI based on the expression of the three genes. The prognostic signature related to autophagy may offer broad prospects for modifying clinical management strategies or at least to prolong the lifespan of HCC patients in the precision medicine era. Due to such evidence, PI may also provide a better program for patients treated with a combination of multiply targeted agents.

Our novel risk score model was constructed and displayed the ability to stratify patients in different pathological stages and histological grades into subgroups with two distinct survival outcomes. More importantly, the predictive model was confirmed by GEO datasets. The TCGA program, other large-scale data and IHC results provided a comprehensive and reliable source to assess the molecular features which were most interrelated with the clinical outcomes of HCC patients. In our previous articles, Wang ZH *et al.* [40] mined RNA-Seq data of HCC patients form the TCGA program and managed to construct a four-lncRNA signature, which was significantly associated with HCC prognosis. Similarly, Li B *et al.* [41] proposed a three-gene predictive signature BASED ON the expression data of three genes (UPB1, SOCS2 and RTN3). However, both these investigations rely on the TCGA dataset and were unable to validate the efficiency by USING other cohorts. Furthermore, we analyzed from the perspective of autophagy, which could offer more precise information in clinical management. As such, our PI model provided potential directions for survival estimates of HCC and future clinical practice may come in the foreseeable future.

Moreover, GSEA was performed to analyze the coordinate expression of genes between the high- and low-risk group. The hallmarks of high-risk were distinguished from low-risk group, by the E2F targets, MYC targets and G2M checkpoint, which revealed that the prognostic signature might be inclined to sustain chronic proliferation. These hallmarks of the high-risk group fully indicated that the proliferative signals were relatively disturbing. Arguably, the ability to proliferate outside of cell cycle control is a fundamental trait of cancer cells [42]. Tumors are prone to autophagy because of the important role of autophagy in supplying amino acids and fatty acids to meet the needs the survival and proliferation needs of the cell [14].

Thus, in conclusion, we assessed the gene expression profiles data of autophagy-related genes based on the TCGA database and proposed a risk score model which had a moderate efficacy in predicting the OS of HCC patients both in univariate and multivariate survival analysis. These results shown that the autophagy-related genes signature is a promising prognostic indictor and provided a better understanding of autophagy in HCC. However, prospective studies are needed to further verify the clinical utility as well as the biological function of the signature using more experiments.

## METHODS

### Differently expressed autophagy-related genes

We obtained autophagy-related genes via database HADb. RNA-seq data of autophagy-related genes from each individual and their clinical information was download from the available TCGA database, which containing 374 HCC and 50 adjacent non-tumor tissues. To test for differential expression between HCC and their non-tumor counterparts, the R package edgeR program was analyzed raw count data with the criteria of Padj < 0.05 and the log FC>1. Then, all the data were converted into [log2 (data+1)] for further analysis.

### Gene functional annotation

To reveal the biological function and disturbing pathways of these differently expressed autophagy-related genes, we used clusterProfiler package of R software for GO, DO and KEGG pathways [43]. The visualize enrichment maps of annotation analysis results were drawn by R with the “ggplot2” and “GOplot” packages.

### Manufacture of potential prognostic signature for HCC

To identify the prognostic genes, these patients were analyzed by univariate Cox proportional hazards regression methods based on expression data as well as the clinicopathological features obtained from TCGA. The procedure was analyzed by using Survival package in R. All statistically significant indicators were selected as candidates for multivariate Cox regression analysis and used to construct the PI score model. The coefficient of each prognostic genes in the risk score model was derived from the corresponding multivariate analysis of these identified indicators. All patients were divided into high-risk or low-risk group based on the median value of the PI, which was calculated according to the expression profiles of the autophagy-related genes and the estimated regression coefficient. PI = exp_BIRC5_*β_BIRC5_ +exp_FOXO1_*β_FOXO1_ +exp_SQSTM1_ *Β_SQSTM1_.

### Verification of the PI via GEO datasets

The online multi-gene biomarkers validation tool SurvExpress (http://bioinformatica.mty.itesm.mx:8080/Biomatec/SurvivaX.jsp) is a gene expression database that includes datasets collected form TCGA and GEO with clinical information. SurvExpress is an online bioinformatics tool to validate the prognostic performance based on a set of genes in various human cancers [44]. Through SurvExpress, we facilitated a Cox regression analysis and separated HCC patients into high- and low- risk groups based on three liver cancer-related datasets: GSE10143, GSE10186 and GSE17856.

### Validation for the expression values of genes

HCC-related microarray expression datasets were searched and downloaded from GEO. A computer-aided microarray search was performed by using the following search terms: (malignan* OR cancer OR tumor OR tumour OR neoplas* OR carcinoma) AND (hepatocellular OR liver OR hepatic OR HCC). The eligible datasets were required to match the following criteria: (1) human tissues; (2) proven diagnosis of HCC; (3) measuring the expression of genes included in PI in HCC and their normal counterparts; (4) providing at least three cases of HCC samples. These datasets were used to determine the expression pattern of the genes included in the PI model between HCC and non-tumor tissues. All the relevant data were extracted and presented as the mean ± SD. To give a comprehensive and visual display of our results, integrated analytic approach was implemented in the form of a meta-analysis. SMD was applied as the effect quantity to evaluate the association between the gene expression levels and HCC using the software STATA 12.0 software. SMD > 0 and its 95% CI not crossing the integer 0 suggested that genes are significantly upregulated in HCC. When SMD < 0 and its 95% CI not crossing the integer 0, it indicated genes are significantly downregulated in HCC. We chose a fixed-effect model or random-effect model to pool the SMD across the GEO datasets depending on the Q statistical analysis. What’s more, we also observed the protein expression pattern of the three genes via The Human Protein Atlas detected by immunohistochemistry.

### Immunohistochemistry

Two tissue microarrays obtained from Pantomics, Inc. (Richmond, CA) containing a total of 302 HCC samples and 11 non-tumor samples were used for the analysis of the present study. We also collected 30 non-tumor tissues from the First Affiliated Hospital of Guangxi Medical University, People’s Republic of China from January 2016 to November 2017. All the collected tissues are treated under the strict confidentiality and according to appropriate applicable laws that protect the confidentiality of personal information. IHC analysis was performed according to the procedure in the manufacturer’s instruction. The polyclonal rabbit anti-SQSTM1 primary antibody (1:1000 dilution; Abcam, ab207305), monoclonal rabbit anti-SQSTM1 primary antibody (1:300 dilution; Abcam, ab52857) and Rabbit anti-Survivin Monoclonal Antibody (1:80 dilution; ZSGB-BIO, ZA-0530) were used in the IHC analysis. Two pathologists (Yi-wu Dang and Gang Chen) independently evaluated the expression level via the IRS. IRS = (staining intensity) * (percentage of marked tumor cells). For percentage of marked tumor cells: 1–10% positive cells scored 1; 11–50% scored 2; 51–80% scored 3; and >80% positive cells scored 4. Staining intensity was scored as: negative staining (0), weak staining (1), moderate staining (2) and strong staining (3). All of the HCC samples were then divided into positive group (IRS ≥ 6) or negative group (IRS < 6) based on the IRS.

### Gene set enrichment analysis

To reveal the meaning of the biological states from the obtained gene expression data between high- and low- risk group, a GSEA (v3.0, Broad Institute, Cambridge, USA) was performed. The phenotype label was set to high-risk versus low-risk. One thousand permutations were conducted to calculate for each analysis. And enrichment map was used for the visualization of the GSEA results. The false discovery rate (FDR) value and normalized enrichment score (NES) were used to identify the hallmarks significantly enriched in each phenotype.

### Statistical analysis

All the statistical analyses were conducted using SPSS 22.0 (Chicago, IL, USA) and STATA 12.0 software (StataCorp, College Station, TX, USA). We used R software, OriginPro 2017 software (Northampton, Massachusetts, USA) and GraphPad Prism 5 (San Diego, CA, USA) for diagram drawing. Independent sample *t*-tests were used to analyze the expression patterns of genes in HCC and the clinicopathological parameters. The survival curves were plotted by Kaplan–Meier (K-M) method, and differences in the survival rates were assessed using the log-rank test. Univariate and multivariate Cox regression analyses were also performed to analyze the independent prognostic value by Survival package of R. All the statistical results were considered to be significant when the *p* value was less than 0.05.

## SUPPLEMENTARY MATERIALS TABLE



## Abbreviations

BP: biological process
CC: cellular component
CI: confidence interval
DAVID: Database for Annotation, Visualization and Integrated Discovery
DO: Disease ontology
GC: gastric cancer
GEO: Gene Expression Omnibus
GO: gene ontology
GSEA: Gene set enrichment analysis
HADb: Human Autophagy Database
HCC: hepatocellular carcinoma
KEGG: Kyoto Encyclopedia of Genes
MF: molecular function
OS: overall survival
PI: prognosis index
RNA-seq: RNA sequencing
SMD: standard mean deviation
TCGA: The Cancer Genome Atlas
