# Supplementary material for: An autophagy-related gene expression signature for survival prediction in multiple cohorts of hepatocellular carcinoma patients
Source: Oncotarget. 2018 Jan 9;9(25):17368–95. doi: 10.18632/oncotarget.24089 (PMC5915122; doi:10.18632/oncotarget.24089)
Supplement: Supplementary file 1 [file oncotarget-09-17368-s001.pdf]

An autophagy-related gene expression signature for survival prediction in multiple cohorts of hepatocellular carcinoma patients

SUPPLEMENTARY MATERIALS

Supplementary Table 1: General information of these prognostic genes

Gene symbol	Full name	Also known as	Entrez Gene ID	RefSeq Accession:
BIRC5	Baculoviral IAP Repeat Containing 5	API4; EPR-1; SURVIVIN	332	NM_001012271
FOXO1	Forkhead Box O1	FKH1; FKHR; FOXO1A	2308	NM_002015
SQSTM1	Sequestosome 1	p60; p62; A170; DMRV; OSIL; PDB3; ZIP3; p62B; NADGP; FTDALS3	8878	NM_003900
